# Substrate Inhibition of 5β-Δ^4^-3-Ketosteroid Dehydrogenase in *Sphingobium* sp. Strain Chol11 Acts as Circuit Breaker During Growth With Toxic Bile Salts

**DOI:** 10.3389/fmicb.2021.655312

**Published:** 2021-03-23

**Authors:** Franziska M. Feller, Gina Marke, Steffen L. Drees, Lars Wöhlbrand, Ralf Rabus, Bodo Philipp

**Affiliations:** ^1^Institut für Molekulare Mikrobiologie und Biotechnologie, Westfälische Wilhelms-Universität Münster, Münster, Germany; ^2^Institute for Chemistry and Biology of the Marine Environment (ICBM), Carl von Ossietzky University of Oldenburg, Oldenburg, Germany; ^3^Fraunhofer-Institut für Molekularbiologie und Angewandte Oekologie IME, Schmallenberg, Germany

**Keywords:** bacterial metabolism, bile acid, biodegradation, dehydrogenase, flavoprotein, steroid

## Abstract

In contrast to many steroid hormones and cholesterol, mammalian bile salts are 5β-steroids, which leads to a bent structure of the steroid core. Bile salts are surface-active steroids excreted into the environment in large amounts, where they are subject to bacterial degradation. Bacterial steroid degradation is initiated by the oxidation of the A-ring leading to canonical Δ^4^-3-keto steroids with a double bond in the A-ring. For 5β-bile salts, this Δ^4^-double bond is introduced into 3-keto-bile salts by a 5β-Δ^4^-ketosteroid dehydrogenase (5β-Δ^4^-KSTD). With the Nov2c019 protein from bile-salt degrading *Sphingobium* sp. strain Chol11, a novel 5β-Δ^4^-KSTD for bile-salt degradation belonging to the Old Yellow Enzyme family was identified and named 5β-Δ^4^-KSTD1. By heterologous production in *Escherichia coli*, 5β-Δ^4^-KSTD function could be shown for 5β-Δ^4^-KSTD1 as well as the homolog CasH from bile-salt degrading *Rhodococcus jostii* RHA1. The deletion mutant of *5β-Δ^4^-kstd1* had a prolonged lag-phase with cholate as sole carbon source and, in accordance with the function of 5β-Δ^4^-KSTD1, showed delayed 3-ketocholate transformation. Purified 5β-Δ^4^-KSTD1 was specific for 5β-steroids in contrast to 5α-steroids and converted steroids with a variety of hydroxy groups regardless of the presence of a side chain. 5β-Δ^4^-KSTD1 showed a relatively low *K*_m_ for 3-ketocholate, a very high specific activity and pronounced substrate inhibition. With respect to the toxicity of bile salts, these kinetic properties indicate that 5β-Δ^4^-KSTD1 can achieve fast detoxification of the detergent character as well as prevention of an overflow of the catabolic pathway in presence of increased bile-salt concentrations.

## Introduction

**Figure 1:**
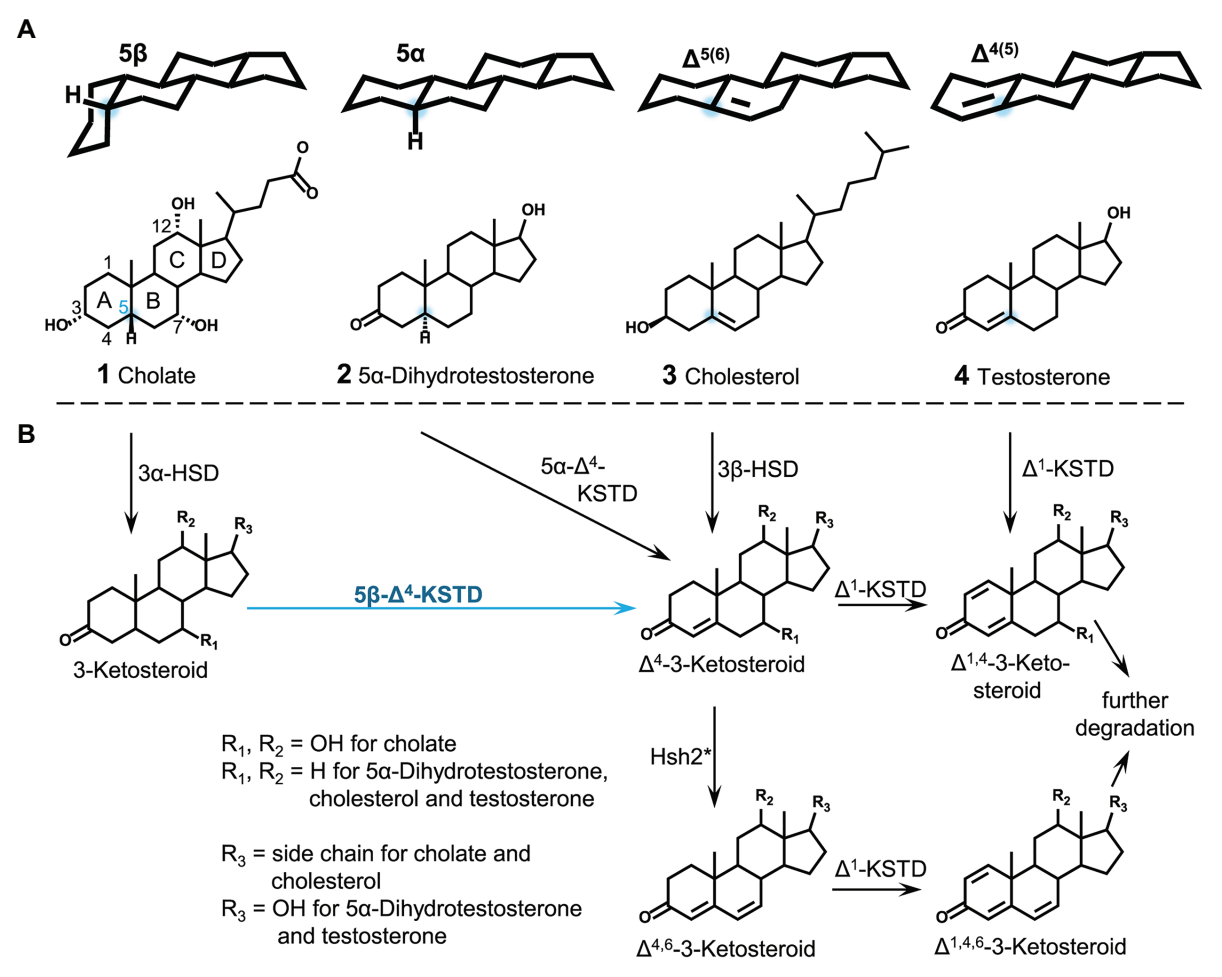
Conformation and structures of steroids from different functional classes with varying stereochemistry at C5 **(A)** and first steps of bacterial steroid degradation, namely A-ring oxidation to canonical Δ^1,4^-3-ketosteroids **(B)**. HSD, Hydroxysteroid dehydrogenase; KSTD, 3-ketosteroid dehydrogenase; Hsh2, hydroxysteroid dehydratase of *Sphingobium* sp. strain Chol11. Blue dots: C5. ^*^This reaction only takes place for 7-hydroxysteroids and in specialized organisms such as *Sphingobium* sp. strain Chol11.

Bile salts such as cholate (**1** in Figure 1) are an important class of steroid compounds in vertebrates that aid in digestion of fatty compounds of food (Hofmann et al., 2010). In general, bile salts have a C_5_ carboxylic side chain attached to C17 of the steroid skeleton and a hydroxyl group at C3; many bile salts also have one or two additional hydroxyl groups attached to C-atoms 6, 7, or 12, respectively. Mammalian bile salts are 5β-steroids with rings A and B in *cis* configuration (Hofmann et al., 2010), which leads to a bent structure of the molecule with all hydroxyl groups on one side (α-side), thereby creating an amphiphilic molecule with detergent properties (Figure 1A). By excretion, large amounts of bile salts are released into the environment, for example, up to about 0.4–0.6 g bile salts per day by each human (Ridlon et al., 2006). In the environment, bile salts are carbon‐ and electron-rich substrates for heterotrophic bacteria such as *Rhodococcus jostii* RHA1, *Pseudomonas stutzeri* Chol1 as well as *Sphingobium* sp. strain Chol11, formerly *Novosphingobium* sp. strain Chol11 (Philipp et al., 2006; Philipp, 2011; Horinouchi et al., 2012; Mohn et al., 2012; Holert et al., 2014; Bergstrand et al., 2016).

As detergents, bile salts are highly toxic for bacteria. Exposition leads to damage of cell membranes, proteins, and DNA (Helenius and Simons, 1975; Begley et al., 2005; Merritt and Donaldson, 2009; Cremers et al., 2014). Therefore, bacteria degrading or transforming bile salts must be able to protect themselves against these toxic effects (Gunn, 2000; Philipp et al., 2006). While degradation itself serves as a detoxification mechanism, bile-salt degrading bacteria must inevitably expose themselves to these compounds as they have to be taken up prior to enzymatic degradation. This dilemma calls for specific strategies when using these toxic detergents as a growth substrate. On the one hand, efflux pumps could be employed to maintain a low intracellular level of bile salts. These are found in many bile-salt resistant intestinal bacteria such as *Escherichia coli* (Thanassi et al., 1997; Gunn, 2000; Venter et al., 2015), but have not been proven in bile-salt degrading bacteria so far.

**Figure 2:**
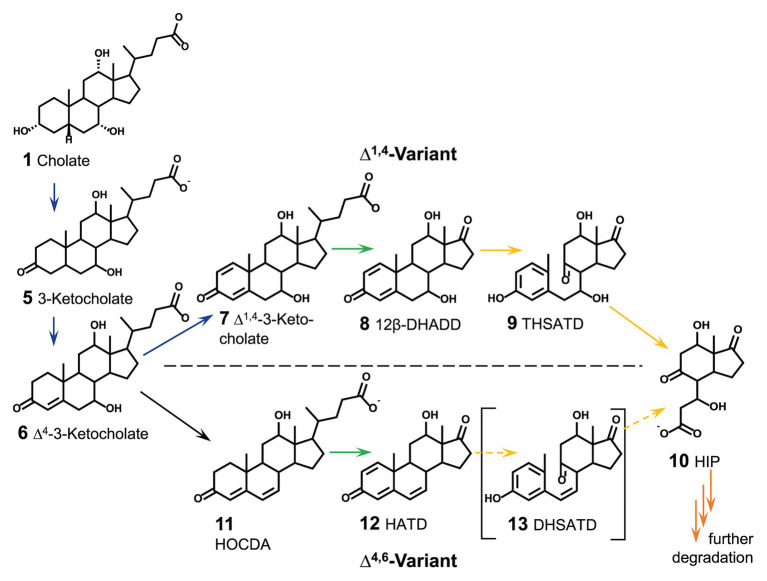
Section of aerobic steroid degradation of model substrate cholate *via* the Δ^1,4^-variant and Δ^4,6^-variant of the 9,10-*seco*-pathway as found in *Pseudomonas stutzeri* Chol1 and *Sphingobium* sp. strain Chol11, respectively. Continuous arrows indicate known reactions, dashed arrows indicate predicted reactions due to bioinformatic analyses, but not experimentally verified. Colors of arrows indicate section of steroid degradation: blue A-ring oxidation, green side-chain degradation, yellow B-ring cleavage and degradation of rings A and B, orange degradation of rings C and D. 12β-DHADD, 7α,12β-dihydroxy-androsta-1,4-diene-3,17-dione; THSATD, 3,7,12-trihydroxy-9,10-*seco*-androsta-1,3,5-triene-9,17-dione; 3αOH-HIP, 3aα-OH-4a(30-propanoate)-7aβ-methylhexahydro-1-indanone; HOCDA, 12-hydroxy-3-oxo-chola-4,6-dienoate; HATD, 12β-hydroxy-androsta-1,4,6-triene-3,17-dione; DHSATD, 3,12-dihydroxy-9,10-*seco*-androsta-1,3,5,6-tetraene-9,17-dione.

**Figure 3:**
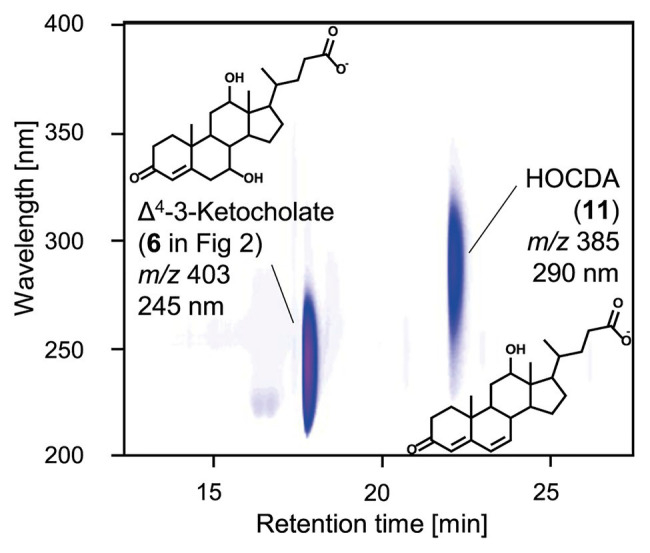
3D-UV-chromatogram showing Δ^4^-3-ketocholate (**6** in Figure 2) and HOCDA (**11**) in an enzyme assay with 3-ketocholate as substrate, cell extracts of *Escherichia coli* MG1655 pBBR1MCS-5::*5β-Δ^4^-kstd1* as well as *E. coli* MG1655 pBBR1MCS-5::*hsh2* and K_3_Fe(CN)_6_ as electron acceptor. Samples were analyzed by high-performance liquid chromatography coupled to mass spectrometry (HPLC-MS) and steroid compounds were identified based on retention time, absorbance spectrum, and mass. Masses are indicated for the respective deprotonated acids.

**Figure 4:**
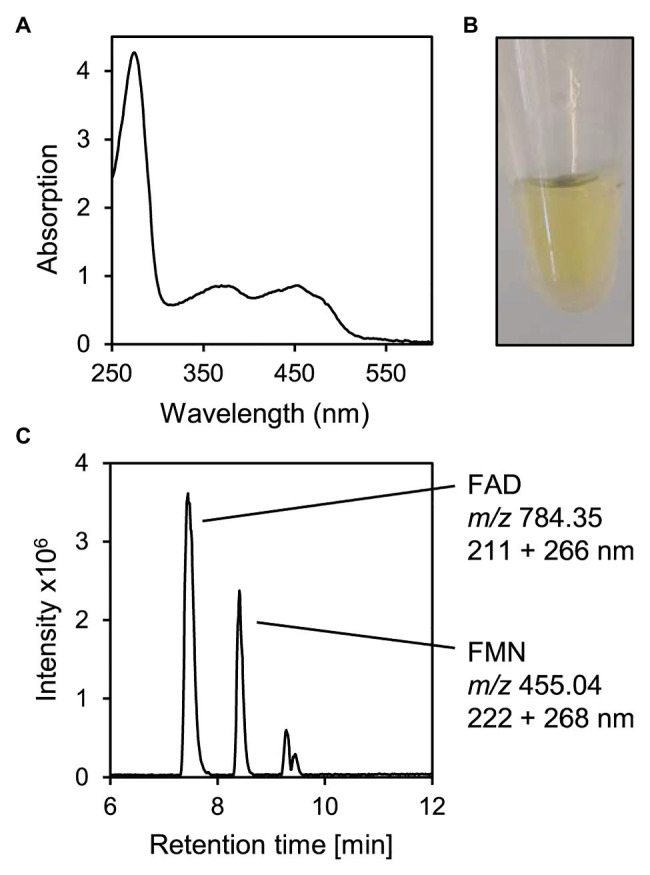
Characteristics of purified 5β-Δ^4^-KSTD1. **(A)** UV/VIS-Absorbance spectrum. **(B)** Yellow-colored solution of purified 2.48 mg ml^−1^ 5β-Δ^4^-KSTD1. **(C)** MS base peak chromatogram of the supernatant of 5β-Δ^4^-KSTD1 denatured with 50% methanol indicating FAD and FMN as cofactors. During HPLC-MS measurements, absorption was measured for 200–320 nm.

On the other hand, the enzymatic transformation of bile salts should quickly abrogate the detergent character. In contrast to the limited knowledge about proteins for bile-salt efflux (and uptake) in bile-salt degrading bacteria, many of the enzymes involved in the respective catabolic pathways for bile-salt degradation are known. Bacterial degradation of bile salts proceeds aerobically *via* the so-called 9,10-*seco*-pathway (Figure 2; Philipp et al., 2006; Philipp, 2011; Mohn et al., 2012) and follows a general scheme (Philipp, 2011; Horinouchi et al., 2012; Wipperman et al., 2014; Olivera and Luengo, 2019): (1) oxidation of the A-ring to Δ^1,4^-3-keto-structures with two double bonds in the A-ring (blue in Figure 2), (2) degradation of the side chain similar to β-oxidation (green in Figure 2), (3) cleavage of rings A and B by oxygenation (yellow in Figure 2), and (4) hydrolytic degradation of the remaining rings C and D (orange in Figure 2). In the well elucidated Δ^1,4^-variant of this pathway, as occurring, e.g., in *P. stutzeri* Chol1, Δ^1,4^-3-ketosteroids are the substrates for ring cleavage (Bergstrand et al., 2016). In contrast, a variation for 7-hydroxy-bile salts *via* Δ^4,6^-ketosteroids and subsequently Δ^1,4,6^-3-ketosteroids is found in α-proteobacteria belonging to the Sphingomonads, which has mainly been investigated in *Sphingobium* sp. strain Chol11 (Holert et al., 2014; Figure 2). *Sphingobium* sp. strain Chol11 was isolated from freshwater using the Δ^4,6^-3-keto-bile salt 12-hydroxy-3-oxo-chol-4,6-dienoate (HOCDA, **11** in Figure 2) as a substrate. The strain is able to degrade various bile salts but is limited in utilization of non-steroidal carbon sources (Yücel et al., 2016). In both degradation variants for 7-hydroxy bile salts, cholate is first oxidized to Δ^4^-3-ketocholate (**6** in Figure 2). In contrast to the introduction of a second double bond in the A-ring in the Δ^1,4^-variation, in the Δ^4,6^-variation water is eliminated from the B-ring by 7α-hydroxysteroid dehydratase Hsh2. This results in the introduction of a double bond at C6 in HOCDA (**11** in Figure 2; Yücel et al., 2016). HOCDA is further degraded to a Δ^1,4,6^-3-keto-C_17_-steroid (**12**), but the mechanisms of side chain degradation and ring cleavage have not been elucidated yet in *Sphingobium* sp. strain Chol11.

Generation of Δ^4^-double bonds in steroid molecules is achieved by different reactions depending on the substrate (Figure 1B). In steroids having a double bond at C5 (such as cholesterol, **3** in Figure 1), the Δ^4^ double bond is introduced by isomerization of the C5 double bond by a combined hydroxysteroid dehydrogenase (HSD) and isomerase (Yang et al., 2007; Uhía et al., 2011; Kreit, 2017). In steroids having no double bond in the A‐ or B-ring Δ^4^-double bonds are introduced by Δ^4^-ketosteroid dehydrogenases (Δ^4^-KSTDs). These enzymes are divided into 5α-Δ^4^-ketosteroid dehydrogenase and 5β-Δ^4^-ketosteroid dehydrogenases (5β-Δ^4^-KSTDs), acting on steroid skeletons with a planar 5α structure [such as dihydrotestosterone (**2** in Figure 1)] vs. bent 5β-structure [such as cholate (**1** in Figure 1)]. Various 5α-Δ^4^-ketosteroid dehydrogenases have been described (Dijkstra et al., 2016): KstD4 from *Rhodococcus erythropolis* SQ1, KstD from *R. jostii* RHA1 (van Oosterwijk et al., 2012) and TesI from *Comamonas testosteroni* (Florin et al., 1996).

Most bile salts in mammals and other vertebrates such as snakes as well as bile alcohols in cartilaginous fish are 5β-steroids (Hofmann et al., 2010). 5β-conformation can also be found in the ecdysteroid-type insect hormones and in steroidal toxins such as digitoxin or batrachotoxin; a further source of 5β-steroids in the environment is coprostanol, the product of microbial cholesterol transformation in many animals and humans (Bull et al., 2002; Gérard, 2013). So far, only one 5β-Δ^4^-KSTD has been purified and characterized from a steroid-degrading bacterium in 1966 (Davidson and Talalay, 1966). This flavoenzyme from *C. testosteroni* has been described to oxidize 5β-3-ketosteroids without or with a C_2_ side chain, while 3-keto-bile salts have not been tested as substrates. No sequence of this protein is known. Thus, no homologs from further bile-salt degrading bacteria could be predicted or found. Two enzymes with 5β-Δ^4^-KSTD activity toward 3-keto-bile salts have been described from *Clostridium scindens* (Kang et al., 2008; Funabashi et al., 2020), that transforms but does not degrade bile salts in the intestine of humans. During this so-called reductive dehydroxylation, hydroxy groups are removed from C7. One step of this process is the introduction of a Δ^4^ double bond (Ridlon et al., 2006). BaiCD and BaiH from *C. scindens* have been described to catalyze this reaction with specificity for 7α-hydroxy bile salts such as cholate or chenodeoxycholate and 7β-hydroxy bile salts such as ursodeoxycholate, respectively (Kang et al., 2008). Although 5β-Δ^4^-KSTD activity can easily be measured in cell extracts of bile-salt degrading bacteria *P. stutzeri* Chol1 (Birkenmaier et al., 2007) and *Sphingobium* sp. strain Chol11 (Holert et al., 2016) with artificial electron acceptors, no 5β-Δ^4^-KSTDs for bile salts that are involved in the first steps of steroid degradation and, thus, bile-salt detoxification, have been found and characterized so far. Therefore, our goal was to identify this enzyme in bile-salt degrading *Sphingobium* sp. strain Chol11 through proteome analysis and to study its role in bile-salt detoxification.

## Materials and Methods

### Cultivation of Bacteria

Strains and plasmids used in this study are listed in Table 1. Strains of *Sphingobium* sp. strain Chol11 (DSM 110934; Holert et al., 2014), *P. stutzeri* Chol1 (DSM 103613; Philipp et al., 2006), *Pseudomonas putida* KT2440 (DSM 6125; Bagdasarian et al., 1981), *R. jostii* RHA1 (McLeod et al., 2006), and *Escherichia coli* MG1655 (DSM 18039; Blattner et al., 1997) were grown in the HEPES buffered mineral medium B (MB) as described previously (Jagmann et al., 2010; Holert et al., 2014). *E. coli* ST18 (DSM 22074; Thoma and Schobert, 2009) and *E. coli* Tuner(DE3) (Novagen, Merck, Darmstadt, Germany) were cultivated in lysogeny broth medium (LB; Bertani, 1951). Whereas 3-ketobile salt oxidation was tested in bile-salt degrading strains *Sphingobium* sp. strain Chol11, *P. stutzeri* Chol1 and *R. jostii* RHA1, *E. coli* MG1655 was used for heterologous expression of untagged putative 5β-Δ^4^-KSTDs and *E. coli* Tuner was used for production of his-tagged 5β-Δ^4^-KSTD for purification. 5-aminolevulinic-acid auxotrophic strain *E. coli* ST18 was used for transferring plasmids to the other strains by biparental conjugation. A strain of *P. putida* KT2440 was used for production of steroid compounds.

**Table 1:** Strains and plasmids used in this study. Cm^r^, Ap^r^, Gn^r^, and Kn^r^: resistance against chloramphenicol, ampicillin, gentamicin, or kanamycin, respectively.

Strain	Description	References
*Sphingobium* sp. strain Chol11	Bile-salt degrading strain	Holert et al., 2014, DSM 110934
Strain Chol11 Δ*5β-Δ^4^-kstd1*	Deletion mutant of *5β-Δ^4^-kstd1*	This study
*P. stutzeri* Chol1	Bile-salt degrading strain	Philipp et al., 2006, DSM 103613
Strain Chol1 Δ*c211_11427*	Deletion mutant of *c211_11427*	This study
*R. jostii* RHA1	Bile-salt degrading strain	McLeod et al., 2006
*P. putida* KT2440	Production of bile-salt derivatives	Bagdasarian et al., 1981, DSM 6125
*E. coli* ST18	Cloning, 5-Ala auxotrophic, *pro thi hsdR^+^* Tpr Smr; chromosome::RP4-2 Tc::Mu-Kan::Tn7/λpir Δ*hemA*	Thoma and Schobert, 2009, DSM 22074
*E. coli* MG1655	Expression and testing of various candidate genes, prototrophic, F^−^, lambda^−^, rph-1	Blattner et al., 1997, DSM 18039
*E. coli* Tuner(DE3)	Protein production, ^−^ *ompT hsdS*_B_ (r_B_^−^ m_B_^−^) *gal dcm lacY1*(DE3)	Novagen
**Plasmid**	**Characteristics**	**References**
pDM4	Gene replacement vector, Cm^r^, *sacB*	Milton et al., 1996
pEX18AP	Gene replacement vector, Ap^r^, *sacB*	Hoang et al., 1998
pBBR1MCS-5	Gene expression vector, Gn^r^	Kovach et al., 1995
pET28B(+)	Gene expression vector, Kn^r^	Novagen

For strain maintenance, wild type strains of *P. stutzeri* Chol1, *Sphingobium* sp. strain Chol11 and *R. jostii* RHA1 were grown with 1 mM cholate as carbon source, whereas deletion mutants of *Sphingobium* sp. strain Chol11 and *E. coli* MG1655 were grown with 15 mM glucose, and deletion mutants of *P. stutzeri* Chol1 as well as *P. putida* KT2440 were grown with 12 mM succinate. In case of pre-cultures and main cultures for cell suspensions of *Sphingobium* sp. strain Chol11 and *E. coli* MG1655 strains, glucose was added, whereas succinate was added for the respective cultures of *P. stutzeri* Chol1 and *P. putida* KT2440 strains.

For cultivating *E. coli* ST18, 50 μg ml^−1^ 5-aminolevulinic acid were added. For cultivation of strains containing pBBR1MCS-5 20 μg ml^−1^ gentamicin were added, when bile salts were added and in main cultures of growth experiments, gentamicin was omitted. For cultivation of strains containing pDM4, 30–90 μg ml^−1^ chloramphenicol were added. *Escherichia coli* strains containing pEX18AP were grown in presence of 100 μg ml^−1^ ampicillin, whereas *P. stutzeri* strains containing pEX18AP were grown in presence of 100 μg ml^−1^ carbenicillin. Strains containing pET28B(+) were cultivated with 50 μg ml^−1^ kanamycin.

For agar plates, 1.5% (w/v) Bacto agar (BD, Sparks, United States) was added to the respective media. Liquid cultures in test tubes or Erlenmeyer flasks were incubated at 30°C and 200 or 120 rpm, respectively, and plates were incubated at 37°C for strains of *E. coli* and 30°C for all other strains.

### Growth Experiments

For growth experiments, 5 ml pre-cultures in 10 ml test tubes were inoculated from agar plates and incubated overnight for about 20 h. Pre-cultures were used to inoculate 3–5 ml medium in 10 ml test tubes with various carbon sources as indicated to an optical density at 600 nm (OD_600_) of about 0.2. Optical density was measured at 600 nm (Camspec M107, Spectronic Camspec, United Kingdom) to determine growth. For high-performance liquid chromatography (HPLC)-measurements of supernatants, samples were withdrawn at the given time points.

Experimental procedures for profiling of soluble proteins by means of two-dimensional difference gel electrophoresis (2D DIGE) are described in Supplementary Text S1 in the Supplementary Material.

### Cell Suspension Experiments

For cell suspension experiments with *Sphingobium* sp. strain Chol11 and deletion mutants, pre-cultures were prepared as described above and incubated for about 20 h. About 50 ml main cultures in 500 ml Erlenmeyer flask were inoculated to an OD_600_ = 0.015 and incubated for about 40 h. Cells were harvested in the exponential growth phase by centrifugation for 8 min at 8,000 × *g* and 4°C. After washing with medium without carbon source, cells were resuspended in medium without carbon source and diluted to an OD_600_ of 1. Cell suspensions were divided into 5 ml aliquots in 10 ml test tubes. About 10 μg ml^−1^ chloramphenicol were added to cell suspension as indicated. To start the experiment, 1 mM cholate was added to the suspension and samples for high-performance liquid chromatography coupled to mass spectrometry (HPLC-MS) were withdrawn instantly and at predefined time points during incubation.

For assessing toxicity of cholate, the latter was added to cell suspensions in given concentrations and number of colony forming units (CFU) were determined after 15 and 90 min at 30°C by drop and plate method after decimal dilution as described (Hoben and Somasegaran, 1982; Jagmann et al., 2010).

### Cloning Techniques and Construction of Unmarked Gene Deletions

Cloning techniques were performed as described elsewhere (Holert et al., 2013a; Yücel et al., 2016).

For heterologous expression, genes *5β-Δ^4^-kstd1*, *nov2c085*, and *nov2c314* from *Sphingobium* sp. strain Chol11, *c211_11247*, and *c211_11427* from *P. stutzeri* Chol1 as well as *casH* from *R. jostii* RHA1 were PCR-amplified using the genomic DNA of the respective wild type strain as template and the respective primer pair expfor/exprev (Table 2). The vector pBBR1MCS-5 (Kovach et al., 1995) as well as the amplified gene were processed with the restriction enzymes as described in Table 2 and ligated. Ligation preparations were used for heat shock transformations with *E. coli* ST18 or MG1655. From *E. coli* ST18, plasmids were transferred to *E. coli* MG1655 or *P. putida* KT2440 by conjugation as described for *P. stutzeri* Chol1 (Holert et al., 2013a). As control, empty vector controls of all strains containing only pBBR1MCS-5 without insert were constructed. Transformations and conjugations were verified by PCR using colony material as template and sequencing using M13 primers.

**Table 2:** Primers used for cloning and construction of unmarked gene deletions.

Name	Sequence	Restriction site
expfor_Chol1_3αHSD	TTTTTTGGATCCATGTCCGTTATCGCAATTA	*Bam*HI
exprev_Chol1_3αHSD	TTTTTTGAATTCTCAGAAGACCTTGGTCCGCA	*Eco*RI
expfor_Chol1_c211_11427	TTTTTTTCTCGAGATGCCCGACTATTCGCACCT	*Xho*I
exprev_Chol1_c211_11427	TTTTTTTTCTAGATCAAACCCTCAACGCCACCT	*Xba*I
expfor_RHA1_casH	TTTTTTTAAGCTTATGACGATAGATCTCGACCCGCC	*Hind*III
exprev_RHA1_casH	TTTTTTTTCTAGATCACGGCACGCGAAGCAC	*Xba*I
expfor_Chol11_5β-Δ^4^-kstd1	TTTTTTTCTCGAGATGACTGCTGCCCCTGCCCT	*Xho*I
exprev_Chol11_5β-Δ^4^-kstd1	TTTTTTTTCTAGATCAGCGGCCCATGGCGG	*Xba*I
expfor_Chol11_nov2c085	TTTTTTTCTCGAGATGGCTTACCGCCACCTCCT	*Xho*I
exprev_Chol11_nov2c085	TTTTTTTTCTAGATCATCCGATCGCGGCGACGG	*Xba*I
expfor_Chol11_nov2c314	TTTTTTTCTCGAGATGGAATTGCGCAACCGGAT	*Xho*I
exprev_Chol11_nov2c314	TTTTTTTTCTAGATCACCCGGCTATTGCGTCCA	*Xba*I
purfor_Chol11_5β-Δ^4^-kstd1	TTTTTTTCATATGACTGCTGCCCCTGCCCT	*Nde*I
purrev_Chol11_5β-Δ^4^-kstd1	TTTTTTTCTCGAGTGAGCGGCCCATGGCGG	*Xho*I
upfor_Chol11_5β-Δ^4^-kstd1	TTTTTTTTCTAGAGTAGAGGTGGAGCGGAATC	*Xba*I
uprev_Chol11_5β-Δ^4^-kstd1	TCTGATCTTCAAGGCCAAAG	
dnfor_Chol11_5β-Δ^4^-kstd1	CTTTGGCCTTGAAGATCAGAGAGAGTCTCCTAATTCGCCA	
dnrev_Chol11_5β-Δ^4^-kstd1	TTTTTTTCTCGAGCTACGACAACCCGGTCAACA	*Xho*I
upfor_Chol1_c211_11427	TTTTTTTTCTAGATGGGACGGGACCAAATTACG	*Xba*I
uprev_Chol1_c211_11427	GGTAGGTGGATGAAAAAGAAAGCC	
dnfor_Chol1_c211_11427	TTCTTTTTCATCCACCTACCGTTTCGTTCCTTGCGATGGGG	
dnrev_Chol1_c211_11427	TTTTTTTCTCGAGCGGGTCGAAAAGCTGATCCT	*Xho*I
upfor_Chol1_stdA1	TTTTTTTTCTAGACTCACGCAATCACGCCACTC	*Xba*I
uprev_Chol1_stdA1	CTGCCGTTCCCCTGTCACCA	
dnfor_Chol1_stdA1	TGGTGACAGGGGAACGGCAGCAGCGCCTCACGCAGCGGCG	
dnrev_Chol1_stdA1	TTTTTTTCTCGAGGACGTGGTCCCAGCCAGGCA	*Xho*I
upfor_Chol1_kstd1	TTTTTTTCTAGAACCAACAACTCCAGCCTCG	*Xba*I
uprev_Chol1_kstd1	GTCCGACTCACTTGCCAGGTT	
dnfor_Chol1_kstd1	AACCTGGCAAGTGAGTCGGACGGAGCGACCGATGAATGCACG	
dnrev_Chol1_kstd1	TTTTTTAAGCTTATGGAGGTAACCGAGGCGA	*Hind*III
M13 for (−43)	AGGGTTTTCCCAGTCACGACGTT	
M13 rev (−49)	GAGCGGATAACAATTTCACACAGG	
pDM4_MCS_for	ACTTAACGGCTGACATGGGA	
pDM4_MCS_rev	GCGAAGTGATCTTCCGTCAC	
T7_for	TAATACGACTCACTATAGGG	
T7_rev	TATGCTAGTTATTGCTCAG	
pDM4_backbone_for	AAG ATG TGG CGT GTT ACG GT	
pDM4_backbone_rev	AGG CTC TGG GAG GCA GAA TA	
sacB_for	AGGAGACATGAACGATGAACA	
sacB_rev	TTTTTTTCCCGGGTCGGCATTTTCTTTTGCGTT	

Underlines indicate restriction sites.

Unmarked deletion mutants of *Sphingobium* sp. strain Chol11 and *P. stutzeri* Chol1 were generated as described (Holert et al., 2013a; Yücel et al., 2016) using splicing by overlapping extension (SOE) PCR (Ho et al., 1989) and primer pairs upfor/uprev and dnfor/dnrev for flanking fragment amplification. Insertion of pDM4 (Milton et al., 1996) into the genome was verified by colony PCR using primer pair pDM4_backbone_for/pDM4_backbone_rev. For deletion of *kstD1* in *P. stutzeri* Chol1, instead of pDM4, suicide vector pEX18AP (Hoang et al., 1998) and primer pair sacB_for/sacB_rev were used. Gene deletion was confirmed by colony PCR with primer pair upfor/dnrev and the deletion mutants were isolated by repeated isolation of single colonies and PCR testing. The deletion mutant was tested by PCR using isolated genomic DNA as well as subsequent sequencing (Supplementary Figure S8).

For complementation of *Sphingobium* sp. strain Chol11 Δ*5β-Δ^4^-kstd1*, empty vector pBBR1MCS-5 and complementation vector pBBR1MCS-5::*5β-Δ^4^-kstd1* as described above were transferred from *E. coli* ST18 into *Sphingobium* sp. strain Chol11 wild type and Δ*5β-Δ^4^-kstd1* by conjugation as described elsewhere (Yücel et al., 2016).

### Preparation of Cell Extracts

Cell extracts of *E. coli* MG1655 pBBR1MCS5 empty vector as well as with various inserts were prepared for enzyme assays. Pre-cultures of all strains in LB with 20 μg ml^−1^ gentamicin were incubated at 30°C for about 8 h. About 50 ml LB with 20 μg ml^−1^ gentamicin and 0.2 mM isopropyl-β-D-thiogalactopyranosid (IPTG) in 500 ml Erlenmeyer flasks without baffles were inoculated with pre-culture to an OD_600_ of 0.015 and incubated overnight for about 16 h. Cells were harvested by centrifugation at 8,000 × *g* for 8 min and room temperature and washed with 10 mM MOPS buffer (pH 7.8 with NaOH). Cells were resuspended in 2 ml 50 mM MOPS buffer (pH 7.8) in 15 ml conical centrifugation tubes. Cell disruption was performed on ice by ultrasonication (UP200S, Hielscher Ultrasonics, Teltow, Germany) with amplitude 60% and cycle 0.5 for two times 4 min with a 2 min break. Cell extracts were transferred to 2 ml reaction tubes, and cell debris was removed by centrifugation for 30 min at 25,000 × *g* and 4°C. Supernatants were divided into aliquots and either used directly or stored at −20°C.

### Purification of 5β-Δ^4^-KSTD1

For purification of enzyme 5β-Δ^4^-KSTD1, *5β-Δ^4^-kstd1* was amplified using genomic DNA of *Sphingobium* sp. strain Chol11 wild type as template and primer pair purfor_Chol11_5β-Δ^4^-kstd1/purrev_Chol11_5β-Δ^4^-kstd1 (Table 2). The fragment was cloned into the expression vector pET28b(+) (Novagen, Merck, Darmstadt, Germany) with restriction enzymes (Table 2) and ligation. The ligation product was transferred into *E. coli* ST18 by heat shock transformation and presence of the insert was verified with colony PCR with primer pair T7_for/T7_rev and subsequent sequencing. The plasmid was transferred into *E. coli* Tuner(DE3) by conjugation as described above for *E. coli* MG1655. Correct transfer of the plasmid was confirmed by colony PCR with primer pair T7_for/T7_rev. Cell extracts of *E. coli* Tuner(DE3) pET28B(+)::*5β-Δ^4^-kstd1* were prepared and tested for 5β-Δ^4^-KSTD activity.

For production of 5β-Δ^4^-KSTD1, 2 l LB medium in four 2 L Erlenmeyer flasks without baffles were inoculated from a pre-culture and incubated at 37°C and 120 rpm orbital shaking. At an OD_600_ of about 0.3, 0.2 mM IPTG were added and temperature was reduced to room temperature. After 16 h further incubation, cells were harvested by centrifugation for 15 min at 8,000 × *g* and 4°C, washed with 10 mM MOPS pH 7.8 and resuspended in 30 ml 50 mM MOPS pH 7.8. The cell suspensions were stored at −75°C overnight until cell disruption. Cells were thawed on ice and, after addition of 100 μl 10 mg ml^−1^ lysozyme and 0.05% Triton X100, disrupted by ultrasonication (UP200S, Hielscher Ultrasonics, Teltow, Germany) with amplitude 60% and cycle 0.5 for four times 3 min on ice. The cell extract was centrifuged for 8 min at 7,000 × *g* and 4°C and for additional 59 min at 19,000 × *g* and 4°C to remove cell debris. The supernatant was filtered through a 0.45 μm PVDF syringe filter (Carl Roth, Karlsruhe, Germany). The tagged protein was purified using a 5 ml HisTrap HP column and FPLC (Äkta start, both GE Healthcare, Chicago, IL, United States). The column was equilibrated with 20 mM Tris buffer pH 8 with 200 mM NaCl and 6 mM imidazole. Cell free lysate was loaded onto the column, which was then washed with 15 column volumes of 20 mM Tris buffer pH 8 with 200 mM NaCl and 6 mM imidazole. The tagged protein was eluted by a gradient from 100% 20 mM Tris buffer pH 8 with 200 mM NaCl and 6 mM imidazole to 60% 20 mM Tris buffer pH 8 with 200 mM NaCl and 250 mM imidazole. Protein containing fractions were collected, pooled, and concentrated with a 10 kDa molecular weight cut-off centrifugal concentrator (Vivaspin, Sartorius, Göttingen, Germany). The protein was freed of imidazole by dialysis against in 2 L 20 mM MOPS pH 7.8 at 4°C overnight.

SDS-PAGE for determination of protein purity was performed according to standard procedures as described elsewhere (Ernst et al., 2020). The absorption spectrum of 5β-Δ^4^-KSTD1 was determined with the help of a nanophotometer (Implen, Munich, Germany), and flavin concentration was estimated using an extinction coefficient of 12,000 M^−1^ cm^−1^ (Ghisla et al., 1974). Flavin cofactors of 5β-Δ^4^-KSTD1 were separated from the protein by denaturing the protein in 50% methanol. Denatured protein was removed by centrifugation for 5 min at >16,000 × *g* and room temperature and the supernatant was measured with HPLC-MS.

### Enzyme Assays

Protein concentration was determined with the help of BCA assay kit (Pierce, Thermo Scientific, Rockford, IL, United States).

If not indicated otherwise, enzyme assays with purified enzyme were conducted in 50 mM MOPS buffer, pH 7.8 with NaOH, with 1 mM K_3_Fe(CN)_6_ as electron acceptor and were started by addition of 0.5 mM 3-ketocholate as substrate. For preliminary tests, assays were conducted in polystyrene 1 ml cuvettes (Sarstedt, Nümbrecht, Germany) and contained 25 μg ml^−1^ enzyme 5β-Δ^4^-KSTD1, whereas most other assays if not indicated otherwise were performed in polystyrene 96-well plates with flat bottom (Sarstedt, Nümbrecht, Germany) with 12.5 μg ml^−1^ 5β-Δ^4^-KSTD1 and path length correction after confirming comparability of activities in both systems. Enzyme activity was measured at 436 nm in a spectrophotometer (UV-2600, Shimadzu, Kyoto, Japan) at 30°C for cuvettes and at 450 nm in a heatable microplate reader (EL808, Biotek, Winooski, VT, United States) at 30°C for microplates. Extinction coefficients for K_3_Fe(CN)_6_ were 0.7 cm^−1^ mM^−1^ at 436 nm and 0.262 cm^−1^ mM^−1^ at 450 nm according to literature and own measurements (Singer, 1974; Birkenmaier et al., 2007). For determination of optimal pH, assays were performed with 50 mM Tris buffer adjusted to various pH values with NaOH or HCl or McIlvaine buffer (McIlvaine, 1921) at different pH-values. Fitting of enzyme kinetics according to appropriate models was performed with Excel Solver using a Levenberg-Marquardt fit for a Michealis-Menten kinetic model for substrate inhibition. This model is equivalent to a model of uncompetitive inhibition, in which substrate and inhibitor concentrations are equated (Equation 1). SDs provided for biochemical constants were determined based on 95% confidence intervals of the fitting.

$$v=\frac{v_{\max}\times \left[S\right]}{K_{m}+\left[S\right]\times \left(1+\frac{\left[I\right]}{K_{i}}\right)\;};\left[S\right]=\left[I\right]$$ (1)

Equation (1) used for fitting steady-state kinetics of Δ^4^-5β-KSTD1. Substrate concentration ([*S*]) is also used for inhibitor concentration ([*I*]).

5α-3-Ketolithocholate, 5β-3-ketolithocholate, 5α-androstan-3,17-dione, and 5β-androstan-3,17-dione solutions were prepared in DMSO with a concentration of about 25 mM. In enzyme assays, about 0.5 mM of these substrates was used after confirming that 5β-Δ^4^-KSTD1 was not inhibited by 2% (v/v) DMSO. Assays containing these substrates were performed in 1 ml 50 mM MOPS in 1.5 ml reaction tubes and incubated at 30°C for 30 min. Endpoint samples were measured with HPLC-MS and compared to controls without enzyme. For determination of K_M_ with 5β-androstan-3,17-dione, assays were performed in microplates as described with 4% (v/v) DMSO in all assays after confirming that there was no inhibitory effect of 4% (v/v) on 5β-Δ^4^-KSTD1.

For assays with other electron acceptors, 1 ml assays were performed in 1.5 ml reaction tubes with 0.5 mM 3-ketocholate and 12.5 μg ml^−1^ 5β-Δ^4^-KSTD1. About 1 mM K_3_Fe(CN)_6_, NAD^+^ or NADP^+^, 25 μM phenazine methosulfate (PMS), 100 μM 2,6-dichlorophenolindophenol (DCPIP) or no electron acceptor for O_2_ control were added and assays were incubated at 30°C and 30 min and heat-inactivated before HPLC-MS measurement.

Enzyme assays with 1 mM NADPH or NADH, about 0.2 mM Δ^4^-3-ketocholate and 25 μg ml^−1^ 5β-Δ^4^-KSTD1 were performed and measured by HPLC-MS to check for reversibility of the reaction. Assays with 0.5 mM K_3_Fe(CN)_6_, 0.1 mM 3-ketocholate, and 25 μg ml^−1^ 5β-Δ^4^-KSTD1 were supplemented with surface active substances [cholate, sodium dodecyl sulfate (SDS), or Tween20] in various concentrations to exclude protein inhibition by surface active properties of 3-ketocholate instead of substrate inhibition.

Enzyme assays with cell extracts of *E. coli* MG1655 were performed with 1 mg ml^−1^ total protein and 1 mM K_3_Fe(CN)_6_ in 1 ml reaction volume in 1.5 ml reaction tubes. Cell extracts were pre-incubated with 1 mM cholate for 30 min at 30°C prior to most enzyme assays to prevent the *E. coli*-enzyme catalyzed oxidation of 7-hydroxy group at the substrate 3-ketocholate. To these assays, 0.5 mM 3-ketocholate, 0.25 mM 3-ketolithocholate, or 240 μl biotransformation supernatant containing 3-ketochenodeoxycholate, 3-ketodeoxycholate, or 3-ketoursodeoxycholate were added.

To determine the position of the double bond, cell extract of *E. coli* MG1655 pBBR1MCS-5::*hsh2* was added (0.4 mg ml^−1^) and assays were incubated at 30°C for additional 30 min. All assays were measured by HPLC-MS.

### Preparation of Steroid Compounds

Cholate (≥99%) from ox or sheep bile as well as deoxycholate (≥97%) and 3-β-hydroxy-5α-cholan-24-oate were purchased from Sigma-Aldrich (St. Louis, MO, United States). Chenodeoxycholic acid (≥98%) and ursodeoxycholate were purchased from Carl Roth GmbH + Co. KG (Karlsruhe, Germany) and Fluorochem (Hadfield, United Kingdom), respectively. 3-Ketocholate was purchased from Chemcruz (Santa Cruz Biotechnology, Dallas, TX, United States). 5α-3-Ketolithocholate, 5β-3-ketolithocholate, 5α-androstan-3,17-dione, and 5β-androstan-3,17-dione were purchased from Steraloids (Newport, RI, United States).

3-Ketodeoxycholate, 3-ketochenodeoxycholate, and 3-ketoursodeoxycholate were prepared by biotransformation with *P. putida* KT2440 pBBR1MCS-5::*3αhsd_Chol1_* (Gene *c211_11247* from *P. stutzeri* Chol1, encoding a 3α-HSD, Refseq accession WP_008568627.1) from deoxycholate, chenodeoxycholate, or ursodeoxycholate, respectively. For this purpose, the strain was incubated with 1 mM of the respective bile salt in 5 ml MB medium with additional 24 mM succinate as carbon source at 30°C in reaction tubes for several days, until a significant amount of the respective 3-keto-bile salt as confirmed by HPLC-MS was produced. For enzyme assays, the culture supernatant after centrifugation for 5 min at >16,000 × *g* and ambient temperature was used. Δ^4^-3-Ketocholate was produced by biotransformation of 1 mM cholate by *P. stutzeri* Chol1 Δ*stdA1* Δ*kstD1* (with gene deletions for steroid CoA ligase StdA1, gene number *c211_111402*, Refseq accession WP_008568657.1 and for 3-ketosteroid-Δ^1^-dehydrogenase KstD1, Refseq accession WP_054094687.1) in 50 ml MB with additional 12 mM succinate in 500 ml Erlenmeyer flasks without baffles. Cultures were incubated until cholate was completely transformed into Δ^4^-3-ketocholate. Supernatants were harvested by centrifugation for 8 min at 8,000 × *g* and room temperature and filtered through a 0.2 μm syringe filter before use.

### Analytical Methods

For HPLC-MS measurements of steroids, samples were withdrawn from enzyme assays as well as cultures and centrifuged for 5 min at >16,000 × *g*. Supernatants were either directly used for measurements or stored at −20°C and centrifuged again prior to measurement.

HPLC-MS measurements were conducted with a Dionex Ultimate 3,000 HPLC (ThermoFisher Scientific, Waltham, Massachusetts, United States) with an UV/visible light diode array detector and a coupled ion trap mass spectrometer (Amazon speed, Bruker; Bremen, Germany) with an electro-spray ion source (ESI). HPLC was equipped with a reversed phase C18 column (Eurospher II 100-5, 150 × 3 mm, 5 μm particle size; Knauer, Berlin, Germany). 20 μl of sample were injected, temperature was 25°C and eluents acetonitrile and 10 mM ammonium acetate with 0.1% formic acid were used. A gradient method starting with 0.3 ml min^−1^ 10% acetonitrile for 2 min, increasing to 90% acetonitrile in 22 min, staying 1 min at 90% acetonitrile and returning to 10% acetonitrile in 1 min with 0.4 ml min^−1^ followed by an equilibration of 4 min at 10% acetonitrile and 0.4 ml min^−1^ was used for all measurements. MS was operated in ultra-scan mode in a scan range of 50–1,000 Da with the following settings: dry gas flow 12 L min^−1^ and dry gas temperature 200°C, nebulizer gas 22.5 psi, polarity alternating, end plate offset 500 V, and capillary voltage 4,000 V.

Cholate concentration was determined from base peak chromatogram in negative mode MS measurements and standard curves. Concentrations of steroid degradation intermediates were determined as peak areas in arbitrary units. 3-Ketocholate was quantified from base peak chromatogram in negative mode MS measurements, Δ^4^-3-ketocholate and Δ^1,4^-3-ketocholate were determined from UV chromatograms at 245 nm, whereas HOCDA was quantified from 290 nm UV chromatograms for most experiments and extracted ion chromatograms for *m/z* 385 in negative mode for growth experiments due to interference with an unknown intermediate. For identification of analytes, molecular masses, and UV absorption spectra as well as retention times were consulted.

Estimation of 5β-Δ^4^-KSTD1 physiological quaternary structure was performed by gel filtration over a 10/300 Superdex 200 increase column (GE Life Sciences, Chicago, IL, United States) with a buffer containing 20 mM MOPS, pH 7.8, and 150 mM NaCl. The chromatography was performed with an ÄKTA FPLC unit at a flow of 0.5 ml min^−1^ and detection at 280 nm. Molecular weight calibration was performed using a mixture of thyroglobulin, bovine γ-globulin, chicken ovalbumin, equine myoglobin, and adenosylcobalamin as reference compounds (BioRad, Hercules, CA, United States).

### Bioinformatical Methods

For search of homologs, BLASTp was used (Altschul et al., 1990; Johnson et al., 2008). For search of homologs with known structure or with published features, BLAST search in RCSB PDB (Berman et al., 2002) and paperBLAST (Price and Arkin, 2017) were used. Prediction of protein domains was performed with Interpro (Mitchell et al., 2019). Alignments for phylogenetic trees were calculated in MegaX (Kumar et al., 2018) with ClustalW algorithm (Thompson et al., 1994). Phylogenetic trees were calculated in MegaX using maximum parsimony method and bootstrap validation with 50 repetitions. The presence of an iron-sulfur cluster was predicted with Interpro, comparisons and with metalpredator (Valasatava et al., 2016). Alignments of genomic DNA of wild type and deletion mutants were calculated using Needleman-Wunsch algorithm in the BLAST suite (Needleman and Wunsch, 1970; Johnson et al., 2008).

## Results

### Proteome Analysis Reveals a Candidate for 5β-Δ^4^-KSTD

During proteome analysis of *Sphingobium* sp. strain Chol11, a protein (Nov2c019, UniProt ID UPI000BE3811D) with 28% identity to BaiCD from *C. scindens* VPI12708 (Kang et al., 2008) stood out. This protein is encoded on the chromosome in vicinity to enzymes with predicted function in steroid A-ring oxidation (Yücel et al., 2018b). Quantitative proteome analysis (2D DIGE), revealed a 5.5-fold increased abundance of Nov2c019 during growth of *Sphingobium* sp. strain Chol11 with cholate as compared to glucose-grown cells (Supplementary Tables S1, S2). Nov2c019 had only low similarity (17% identity for each) to known Δ^1^-KSTDs and 5α-Δ^4^-KSTDs such as TesH and TesI, respectively, from *C. testosteroni*.

### Nov2c019 Displays KSTD Activity When Expressed Heterologously

For further elucidation of Nov2c019 function, its gene was expressed from a plasmid in *E. coli* MG1655. In enzyme assays with 3-ketocholate (**5** in Figure 1) as substrate, K_3_Fe(CN)_6_ as electron acceptor and cell extract of *E. coli* MG1655 pBBR1MCS-5::*nov2c019*, a steroid compound 2 Da lighter than 3-ketocholate with a characteristic absorbance at 245 nm indicating a double bond in the A-ring (Holert et al., 2013b) was formed (Figure 3). For determining the position of the double bond, cell extract of *E. coli* MG1655 pBBR1MCS-5::*hsh2* was added. Hsh2 is a 7-hydroxysteroid dehydratase from *Sphingobium* sp. strain Chol11, that stereospecifically catalyzes elimination of water from 7α-hydroxyl groups in bile salts with 3-keto group and a Δ^4^-double bond (Yücel et al., 2016). Upon addition of this cell extract, HOCDA (**11**) was formed as indicated by its characteristic absorption at 290 nm and molecular mass of 385 Da for the deprotonated acid (Holert et al., 2014). This outcome with Hsh2 as a diagnostic tool shows that the first double bond had to be in Δ^4^-position as this is the prerequisite for the formation of Δ^4,6^-3-ketosteroid compounds with this characteristic absorption. Thus, Nov2c019 could catalyze the desaturation of the C−C bond between carbon atoms 4 and 5 in a 5β-steroid and was named 5β-Δ^4^-KSTD1, accordingly.

### Purified 5β-Δ^4^-KSTD1 Efficiently Oxidizes 5β-3-Ketosteroids to Respective Δ^4^-3-Ketosteroids

For further characterization of 5β-Δ^4^-KSTD1, the enzyme was purified. Using his-tags at both C‐ and N-termini due to its large size, the enzyme could be isolated by affinity chromatography (Supplementary Figure S2). Bioinformatic analyses predicted a relatively large protein of about 75 kDa belonging to the family of old yellow enzymes. These analyses also predicted two flavin cofactors as well as an [4Fe-4S] cluster involving cysteines C375, C378, C381, and C393. Consistent with this, a protein with a mass of slightly more than 70 kDa (Supplementary Figure S1), absorbance maxima characteristic for flavin cofactors at about 260 and 450 nm (Figure 4A) and corresponding yellow color (Figure 4B) was purified. A 2-fold higher flavin concentration of about 70 μM compared to protein concentration of 2.48 mg ml^−1^ (corresponding to about 33 μM) indicated a stoichiometry of two flavin cofactors per enzyme molecule. HPLC-MS measurements confirmed that denaturing the enzyme released flavin cofactors, which were identified as FAD and FMN by MS (Figure 4C). Gel filtration of the purified protein showed that mostly molecules with a mass of 70 kDa were present, indicating that 5β-Δ^4^-KSTD1 is a monomeric enzyme (Supplementary Figure S2).

In enzyme assays with K_3_Fe(CN)_6_ as electron acceptor, purified 5β-Δ^4^-KSTD1 completely converted 3-ketocholate (**5** in Figure 2) to Δ^4^-3-ketocholate (**6**; Supplementary Figure S3A), and addition of cell extract containing Hsh2 led to the formation of HOCDA (**11**) again, confirming the product to be Δ^4^-3-ketocholate (Supplementary Figure S3B).

**Figure 5:**
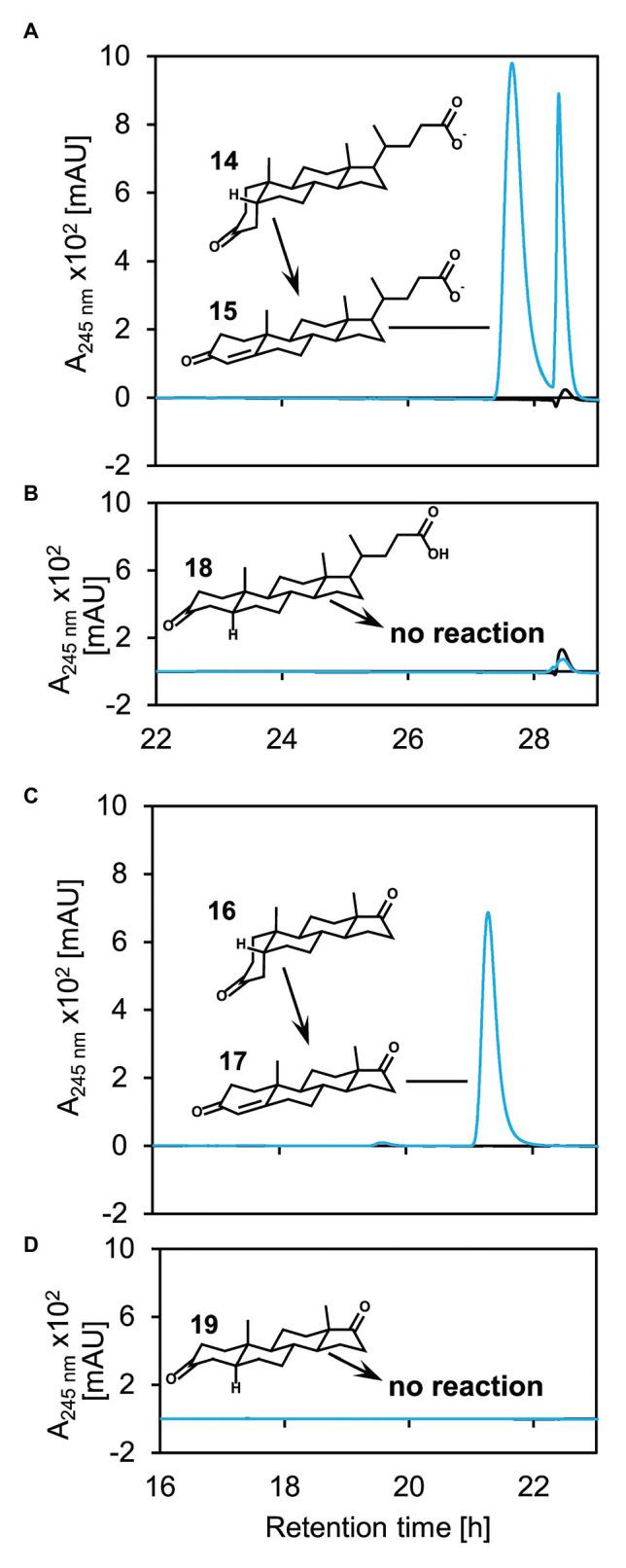
Catalytic activity of 5β-Δ^4^-KSTD1 toward different steroids with (**A**+**B**) or without (**C**+**D**) side chain and 5β‐ (**A**+**C**) or 5α‐ (**B**+**D**) structure. UV chromatograms of enzyme assays with 5β-Δ^4^-KSTD1, K_3_Fe(CN)_6_ as electron acceptor and 5β-3-ketolithocholate (**14**, **A)**, 5α-3-ketolithocholate (**18**, **B)**, 5β-androsta-3,17-dione (**16**, **C)**, or 5α-androsta-3,17-dione (**19**, **D**) as substrates. Blue enzyme assays containing substrate, electron acceptor, and 5β-Δ^4^-KSTD1, black controls without 5β-Δ^4^-KSTD1. The second peak found in the enzyme assays with 5β-3-ketolithocholate **(A)** stems from an unidentified contamination without characteristic absorption maximum at 245 nm. Enzyme assays were analyzed by HPLC-MS and steroid compounds were identified by retention time, absorbance spectrum, and mass. **15**: Δ^4^-3-ketolithocholate, **17**: Δ^4^-androstene-3,17-dione. Both, 5α‐ and 5β-androsta-3,17-dione, as well as 5α and 5β-3-ketolithocholate show no absorption at 245 nm and are therefore not visible in the UV-chromatograms.

For analyzing the substrate spectrum of 5β-Δ^4^-KSTD1, 5α‐ as well as 5β-steroids with and without side chain but with otherwise identical structure were used as substrates in enzyme activity assays (Figure 5). 5β-Δ^4^-KSTD1 was able to oxidize steroids having a 5β-nucleus both, with and without side chain, namely 3-ketolithocholate (**14** in Figure 5A) and 5β-androstane-3,17-dione (**16**; Figure 5C) to Δ^4^-3-ketolithocholate (**15**) and androst-Δ^4^-ene-3,17-dione (**17**), respectively. In contrast, the respective 5α-equivalents 5α-3-ketolithocholate (**18**; Figure 5B), and 5α-androstane-3,17-dione (**19**; Figure 5D) were not converted. In enzyme assays testing different 3-keto-bile salts as potential substrates, 5β-Δ^4^-KSTD1 was able to catalyze oxidation of other 3-keto-bile salts with varying hydroxy groups attached to the steroid nucleus to the respective products Δ^4^-3-ketochenodeoxycholate (**20** in Supplementary Figure S4A), Δ^4^-3-ketodeoxycholate (**21**; Supplementary Figure S4B), and Δ^4^-3-ketoursodeoxycholate (**22**; Supplementary Figure S4C).

Additionally, no reverse reaction reducing Δ^4^-3-ketocholate (**6** in Figure 2) to 3-ketocholate (**5**) could be observed in enzyme assays containing NADH or NADPH as electron donors and Δ^4^-3-ketocholate as substrate (data not shown).

### 5β-Δ^4^-KSTD1 Displays Substrate Inhibition

**Figure 6:**
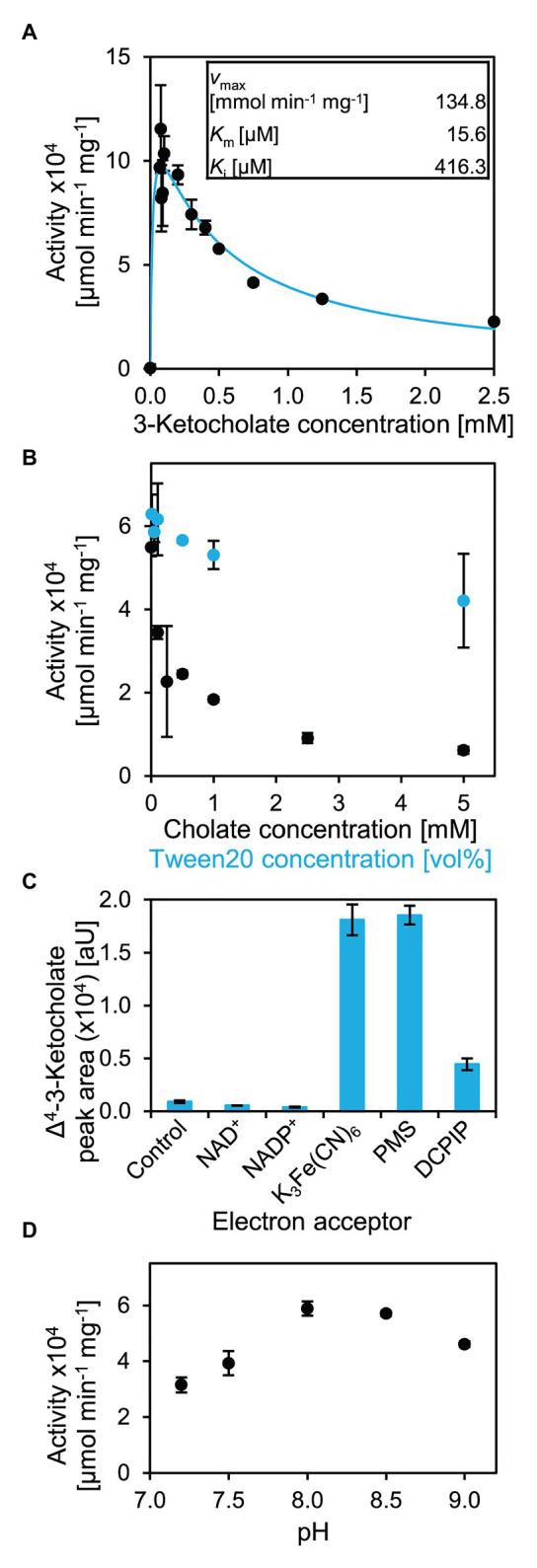
Catalytic properties of 5β-Δ^4^-KSTD1. **(A)** Kinetics of 5β-Δ^4^-KSTD1. Activity with varying concentrations of 3-ketocholate (**5** in Figure 2) and 1 mM K_3_Fe(CN)_6_ at pH 7.8 (black) and modeling of kinetics for substrate inhibition with the given constants (blue). **(B)** Inhibitory effects of detergents cholate (black) and Tween20 (blue) on enzyme activity of 5β-Δ^4^-KSTD1 with 0.2 mM 3-ketocholate and 1 mM K_3_Fe(CN)_6_. **(C)** Specificity of 5β-Δ^4^-KSTD1 regarding electron acceptors. Final concentrations of product Δ^4^-3-ketocholate in enzyme assays with 5β-Δ^4^-KSTD1, 0.5 mM 3-ketocholate and varying electron acceptors. The control contained no electron acceptor besides oxygen from air that is found in all assays. Concentrations of electron acceptors were 1 mM NAD^+^, 1 mM NADP^+^, 1 mM K_3_Fe(CN)_6_, 25 μM PMS, 100 μM DCPIP. Enzyme assays were analyzed by HPLC-MS and steroid compounds were identified by retention time, absorbance spectrum, and mass. **(D)** Influence of pH on enzyme activity of 5β-Δ^4^-KSTD1 with 0.5 mM 3-ketocholate as substrate in Tris-Cl buffer. Error bars indicate SD, which may not be visible if too small (*n* = 3).

By monitoring absorption changes upon K_3_Fe(CN)_6_ reduction, enzyme activity of 5β-Δ^4^-KSTD1 could be determined photometrically. With 1 mM of K_3_Fe(CN)_6_, kinetics showed a maximum activity with about 0.1 mM 3-ketocholate (**5** in Figure 2). Higher concentrations of the organic substrate led to strongly decreasing rates, indicating substrate inhibition (Figure 6A). Indeed, using a Michealis-Menten kinetic model for substrate inhibition, which is equivalent to a model of uncompetitive inhibition, in which substrate and inhibitor concentrations are equated, the data could be described accurately. The Levenberg-Marquardt fit led to a *v*_max_ of about 134.8 ± 50.7 mmol min^−1^ mg^−1^, *K*_m_ = 15.6 ± 32.6 μM and *K*_i_ = 416.3 ± 250.1 μM. Assuming a purity of the substrate of 100%, these parameters result in a *k*_cat_ of 16.9 ± 6.3 × 10^4^ s^−1^. SDs were calculated out of 95% CIs given by the fitting algorithm, which is more accurate than, e.g., averaging the parameters from three replicate fits and calculating the respective SDs therefrom (Sheskin, 2007). A possible reason for the inhibitory effect of 3-ketocholate could be its detergent character that may affect enzyme functionality. We therefore undertook tests to determine the general susceptibility of the enzyme for detergent-induced unspecific inhibition. At a 3-ketocholate concentration of 0.2 mM no activity was detected with 35 μM of SDS (data not shown). However, in the presence of 45 mM [5% (v/v)] of the non-ionic detergent Tween20, activity only dropped by 20% (Figure 6B). Cholate (**1** in Figure 2) showed very strong concentration-dependent enzyme inhibition leading to a loss of about 95% activity with 5 mM cholate and resembling inhibition found with higher 3-ketocholate concentrations (Figure 6B). These data indicate that 5β-Δ^4^-KSTD1 is not suffering from a general susceptibility to surface-active compounds, but besides 3-ketocholate is inhibited by other bile salts.

For analyzing enzyme kinetics with a 5β-steroid without side chain, 5β-androstane-3,17-dione (**16** in Figure 5) was used. However, no increase of activity with increasing substrate concentration could be observed, possibly because substrate saturation is already achieved at concentrations below detection limit of the spectrophotometric assay (i.e., a very low *K*_m_ of 5β-Δ^4^-KSTD1 with 5β-androstane-3,17-dione, Supplementary Figure S5A). The enzyme activity of about 6 mmol min^−1^ mg^−1^ was lower compared to 3-ketocholate, and no substrate inhibition was observed. However, enzyme assays were restricted by low substrate solubility in water and ambiguous concentration determination because of limited purity of the commercial substrate.

For analyzing possible electron acceptors, NAD^+^, NADP^+^, PMS, and DCPIP, as well as a control without further electron acceptor besides atmospheric O_2_ were tested (Figure 6C). In assays containing PMS and K_3_Fe(CN)_6_, 0.5 mM 3-ketocholate (**5** in Figure 2) was completely transformed after 30 min incubation at 30°C. In assays containing DCPIP, this conversion was much less effective and reached only 25% of the conversion with K_3_Fe(CN)_6_. In assays with NAD^+^ or NADP^+^ as electron acceptors, the conversion was even less effective and did not differ from assays in which only oxygen from air could serve as electron acceptor.

Optimal pH for 5β-Δ^4^-KSTD1 was determined to be about 8 (Figure 6D) when using 50 mM Tris-Cl buffer. Consistently, enzyme activity linearly increased with pH from 5.5–8 when using McIlvaine buffer (Supplementary Figure S5B).

### Phenotype of the Unmarked Deletion Mutant of *5β-Δ^4^-kstd1* Shows Reduced Oxidation of 3-Ketocholate to Δ^4^-3-Ketocholate

**Figure 7:**
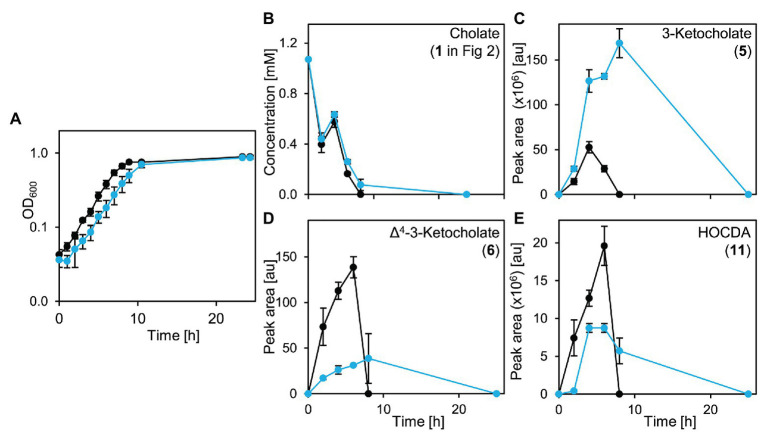
Growth of *Sphingobium* sp. strain Chol11 wt (black) and Δ*5β-Δ^4^-kstd1* (blue) with cholate (**1** in Figure 2; **A**), degradation of cholate (**B**) and accumulation of intermediates 3-ketocholate (**5** in Figure 2, **C**), Δ^4^-3-ketocholate (**6**, **D**) and HOCDA (**11**, **E**) in the cell-free culture supernatant. Error bars indicate SD and may not be visible if too small (*n* = 3).

An unmarked deletion mutant of the corresponding gene, *Sphingobium* sp. strain Chol11 Δ*5β-Δ^4^-kstd1*, was constructed and showed only slightly, but in several biological replicates very consistently delayed growth with cholate (**1** in Figure 2) as sole carbon source with about 1 h lag-phase compared to the wild type without lag phase (Figure 7A). This prolonged lag-phase was not observed when cells were grown with glucose (data not shown). Both strains had similar growth rates and reached a similar final optical OD_600_. Cholate degradation by the deletion mutant and the wild type was very similar (Figure 7B), but regarding the accumulation of degradation intermediates, more pronounced differences were observed. In particular, the accumulation of 3-ketocholate (**5** in Figure 2) was thrice as high in the mutant cultures and lasted longer than in wild type cultures, whereas accumulation of the next intermediates Δ^4^-3-ketocholate (**6**) and HOCDA (**11**) was about 50% lower and set on about 2 h later (Figures 7C–E). Complementation of the deletion mutant with plasmid-borne *5β-Δ^4^-kstd1* yielded no clear growth differences between complemented strain, empty vector control, and respective wild type control strains (data not shown). Probably, burden from bearing of the plasmid interfered with the positive effect of the complementation.

**Figure 8:**
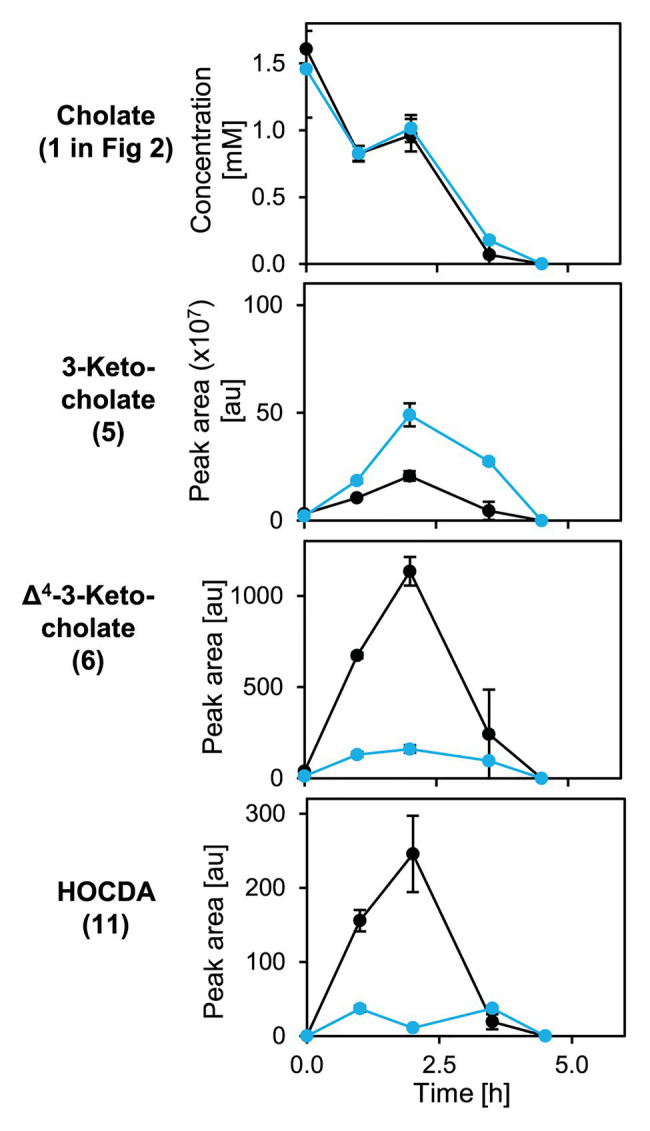
Degradation of cholate (**1** in Figure 2) by suspensions of glucose-grown cells of *Sphingobium* sp. strains Chol11 wt (black) and Δ*5β-Δ^4^-kstd1* (blue; OD_600_ = 1) and transient accumulation of intermediates 3-ketocholate (**5**), Δ^4^-3-keto-cholate (**6**), and HOCDA (**11**) in the cell-free culture supernatant. Error bars indicate SD, which may not be visible if too small (*n* = 3).

**Figure 9:**
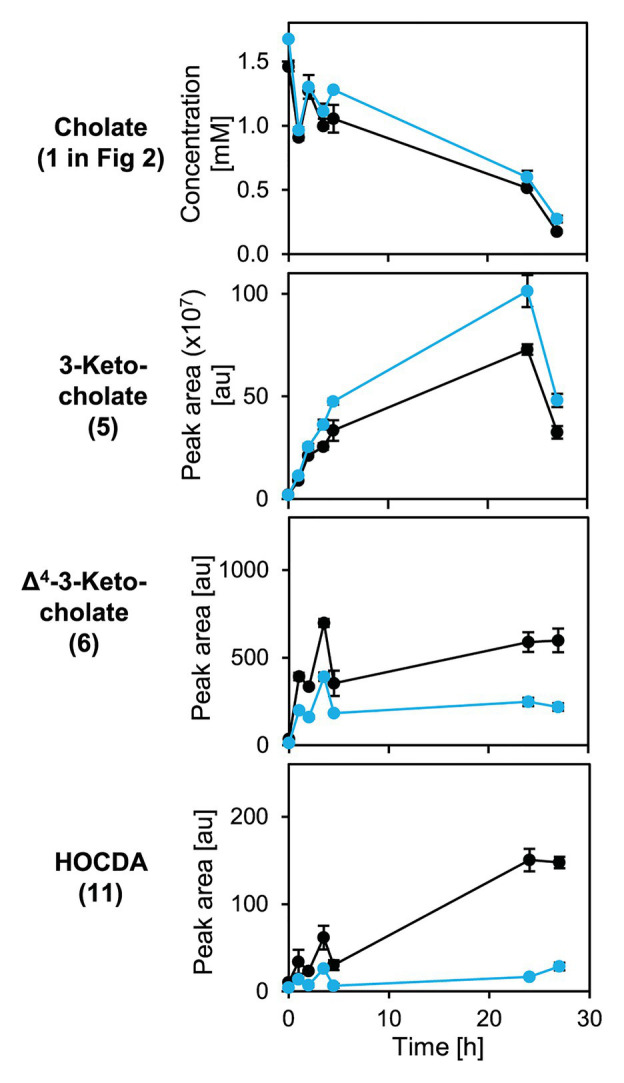
Degradation of cholate (**1** in Figure 2) by suspensions of glucose-grown cells of *Sphingobium* sp. strains Chol11 wt (black) and Δ*5β-Δ^4^-kstd1* (blue; OD_600_ = 1) in presence of chloramphenicol and transient accumulation of intermediates 3-ketocholate (**5**), Δ^4^-3-keto-cholate (**6**), and HOCDA (**11**) in the cell-free culture supernatant. Chloramphenicol was added after preparation of cell suspensions prior to substrate addition. Error bars indicate SD, which may not be visible if too small (*n* = 3).

To analyze cholate degradation and intermediate accumulation in more detail, suspensions of resting glucose-grown cells were supplied with cholate (Figure 8). Here, a similar trend as in growth experiments was observed with comparable cholate degradation in less than 5 h by both strains, enhanced 3-ketocholate (**5** in Figure 2) accumulation and delayed as well as strongly decreased Δ^4^-3-ketocholate (**6**) and HOCDA (**11**) accumulation in mutant cells compared to wild type cells. These results agree with the function as a 5β-Δ^4^-KSTD. However, loss of *5β-Δ^4^-kstd1* could apparently be complemented by other genes. For investigating whether the prolonged lag-phase was due to a delayed upregulation of compensatory genes or lower activity of alternative enzymes, suspensions of glucose-grown cells were incubated with cholate and chloramphenicol (Figure 9). Here, cholate degradation by both strains was much slower with residual cholate detected even after 27 h. In suspensions of both strains, 3-ketocholate accumulated and was not completely degraded after 27 h, but 50% more 3-ketocholate accumulated in cell suspensions of the deletion mutant compared to the wild type. In contrast, Δ^4^-3-ketocholate and HOCDA accumulated about 5-fold more in suspensions of wild type compared to mutant cells indicating that compensatory genes could not be expressed.

As 5β-Δ^4^-KSTD1 seems to be involved in the first steps of cholate degradation, which could also pose a detoxification mechanism of bile salts in *Sphingobium* sp. strain Chol11, the effect of Δ*5β-Δ^4^-kstd1* deletion on cholate sensitivity of *Sphingobium* sp. strain Chol11 was analyzed.

Growth experiments with different cholate concentrations revealed that *Sphingobium* sp. strain Chol11 wild type grew readily with 1 and 2 mM cholate, while growth was inconsistently retarded with 3 mM and completely inhibited with 5 mM (Supplementary Figures S6A,B). With both, 2 and 3 mM cholate, *Sphingobium* sp. strain Chol11 Δ*5β-Δ^4^-kstd1* growth was delayed compared to the wild type with a lag phase of about 2 h (Supplementary Figure S6B). To further investigate cholate sensitivity of *Sphingobium* sp. strain Chol11, remaining CFUs in cell suspensions after incubation with different cholate concentrations were determined (Supplementary Figures S6C,D). However, no significant differences between *Sphingobium* sp. strain Chol11 wild type and Δ*5β-Δ^4^-kstd1* were observed. Whereas 1 mM cholate had no effect on both strains after 15 and 90 min, CFUs in cell suspensions of both strains incubated with 10 mM cholate decreased by a factor of 10 and 100 after 15 and 90 min, respectively. When incubated with 50 mM cholate, CFUs in both cell suspensions decreased by a factor of 10^6^ after 15 min, and no CFUs could be detected after 90 min.

### 5β-Δ^4^-KSTD1 Homologs Are Present in Many Bile-Salt Degrading Bacteria

Principally, bile-salt degradation as found in several bacteria should always involve 5β-Δ^4^-KSTDs as this reaction is required for both the Δ^1,4^‐ and the Δ^4,6^-variant. Therefore, genomes of known bile-salt degrading bacteria were searched for potential homologs of 5β-Δ^4^-KSTD1 (Table 3). In the genome of *Sphingobium* sp. strain Chol11 (RefSeq assembly accession GCF_900218065.1), the existence of isoenzymes was predicted due to the phenotype of the deletion mutant, and indeed two further Old Yellow Enzymes with 46 and 62% identity to 5β-Δ^4^-KSTD1, respectively, were found. However, neither homolog was detected during the proteome studies (unpublished results). In each *P. stutzeri* Chol1, *Azoarcus* sp. strain Aa7, *C. testosteroni* CNB-2, *Dietzia* sp. strain Chol2 as well as *R. jostii* RHA1, which are all known to catalyze 5β-steroid-Δ^4^-dehydrogenation (Philipp et al., 2006; Horinouchi et al., 2012; Mohn et al., 2012; Holert et al., 2014; Yücel et al., 2018a), one respective homolog with identities between 28% for CasH from *R. jostii* RHA1 and 48% for a putative 5β-Δ^4^-KSTD from *Azoarcus* sp. strain Aa7 were found. All potential homologs had similar domain structure belonging to the Old Yellow Enzyme family and had 28–30% identity to BaiCD from *C. scindens* VPI12708. The respective genes coding for CasH from *R. jostii* RHA1 and CtCNB1_1320 from *C. testosteroni* are located in clusters of cholate degradation genes (Horinouchi et al., 2012; Mohn et al., 2012), but no function could be assigned yet (Bergstrand et al., 2016).

**Table 3:** Homologs of 5β-Δ^4^-KSTD1 in several known bile-salt degrading organisms and their identity to 5β-Δ^4^-KSTD1 and BaiCD.

Strain	Protein	UniProt-ID	Identity to (%)	
			5β-Δ^4^-KSTD1	BaiCD
*Sphingobium* sp. strain Chol11	5β-Δ^4^-KSTD1	UPI000BE3811D	100	28
	Nov2c085	UPI000BE23967	46	30
	Nov2c314	UPI000BE376EE	62	28
*Pseudomonas stutzeri* Chol1	c211_11427	K5YL27	44	28
*Comamonas testosteroni* CNB-2	CtCNB1_1320	D0J386	43	29
*Azoarcus* sp. strain Aa7	AZOA_21170	WP_191659088.1^∗^	48	30
*Rhodococcus jostii* RHA1	CasH	Q0S3N3	28	30
*Dietzia* sp. strain Chol2	2,765	NA^∗∗^	41	30
*Clostridium scindens* VPI 12708	BaiCD	P19410	28	100
	BaiH	P32370	29	32

∗ Only RefSeq accession number available so far.

∗∗ For sequence, see Supplementary Figure S7.

**Figure 10:**
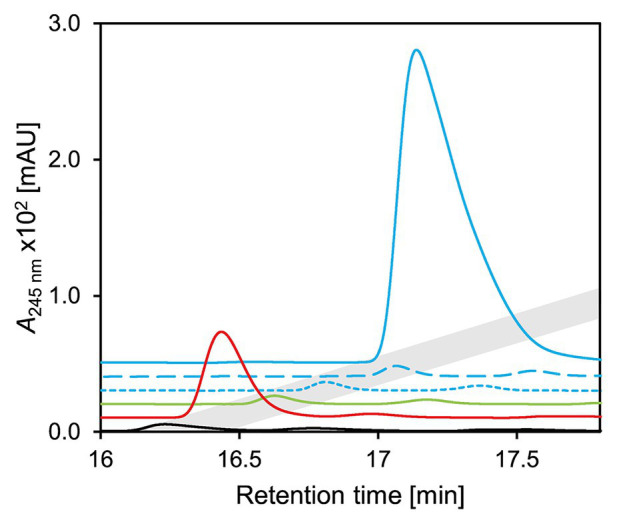
UV-chromatograms of enzyme assays with 3-ketocholate (**5** in Figure 2) as substrate, K_3_Fe(CN)_6_ as electron acceptor and cell extracts of *E. coli* MG1655 pBBR1MCS5 carrying genes for homologs of 5β-Δ^4^-KSTD1. Continuous blue *5β-Δ^4^-kstd1*, dashed blue *nov2c085*, dotted blue *nov2c314*, all from *Sphingobium* sp. strain Chol11; green *c211_11427* from *P. stutzeri* Chol1, red *casH* from *Rhodococcus jostii* RHA1, black empty vector control. Chromatograms are depicted with an offset in both retention time and intensity for easier distinction. Gray indicates retention time of Δ^4^-3-ketocholate (**6**). Enzyme assays were analyzed by HPLC-MS and steroid compounds were identified by their retention time, absorbance‐ and mass-spectrum.

For testing the function of these homologs, selected genes were expressed in *E. coli* MG1655 and the corresponding enzymes were tested in cell extracts with 3-ketocholate (**5** in Figure 1) and K_3_Fe(CN)_6_. CasH from *R. jostii* RHA1, similar to 5β-Δ^4^-KSTD1, was able to oxidize 3-ketocholate to Δ^4^-3-ketocholate (**6**; Figure 10) as well as the bile-salt derivatives 3-ketochenodeoxycholate, 3-ketodeoxycholate, 3-ketoursodeoxycholate, and 3-ketolithocholate with differing hydroxy group substitutions to the respective Δ^4^-3-keto bile salt (**20**–**22** in Supplementary Figure S4 and **14** in Figure 5, respectively; Supplementary Figure S4). In contrast, no activity could be detected in enzyme assays with Nov2c085 and Nov2c314, the homologs from *Sphingobium* sp. strain Chol11, or C211_11427 from *P. stutzeri* Chol1 for all tested bile salts (Figure 10, Supplementary Figure S4). Accordingly, a respective unmarked deletion mutant of *P. stutzeri* Chol1, *P. stutzeri* Chol1 Δ*c211_11427*, did not show an altered phenotype regarding growth with cholate as sole carbon source, although no further homologs could be found in its genome (data not shown).

**Figure 11:**
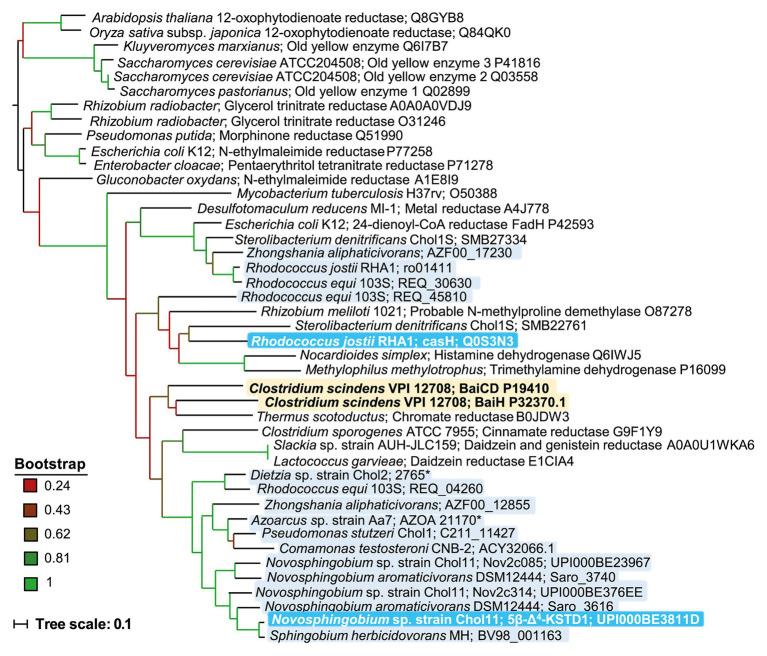
Phylogenetic tree of homologs of 5β-Δ^4^-KSTD1 and further enzymes from the old yellow enzyme family. The tree was constructed based on ClustalW alignment and maximum parsimony method. Branch colors indicate bootstrap values as indicated. Proteins were chosen from BLAST searches among well characterized proteins with verified function or structure or from known bile-salt degrading bacteria. Yellow shading: Enzymes with confirmed 5β-bile-salt Δ^4^-dehydrogenase function in bile-salt dehydroxylating bacteria, light blue shading: enzymes from bile-salt degrading bacteria, dark blue shading: enzymes from bile-salt degrading bacteria with confirmed 5β-bile-salt Δ^4^-dehydrogenase function.

In a phylogenetic tree together with well-characterized enzymes from the Old Yellow Enzyme family, 5β-Δ^4^-KSTD1, CasH as well as BaiCD and BaiH, which were experimentally confirmed as 5β-Δ^4^-KSTDs, formed a cluster together with several other dehydrogenases that can transform complex substrates such as daidzein (Figure 11). Within this cluster, homologs from several bile-salt degrading proteobacteria such as *Sphingomonadaceae*, *P. stutzeri* Chol1, and *C. testosteroni* are found together with 5β-Δ^4^-KSTD1, whereas BaiCD and BaiH as well as CasH seem to be more distant from these. However, in the given selection of proteins, steroid dehydrogenases did not seem to be clustered separately from enzymes with other functions.

## Discussion

In this work, we identified and characterized the flavoenzyme 5β-Δ^4^-KSTD1 catalyzing the conversion of 3-ketocholate (**5** in Figure 2) to Δ^4^-3-ketocholate (**6**) in *Sphingobium* sp. strain Chol11 and analyzed its physiological role for the degradation of toxic bile salts. Apparently, 5β-Δ^4^-KSTD1 is very similar to 5β-Δ^4^-KSTD from *C. testosteroni* that has been purified decades ago and for which no sequence information existed (Davidson and Talalay, 1966).

5β-Δ^4^-KSTD1 is characterized by a very high specific activity and relatively low *K*_m_ compared to most other enzymes (Bar-Even et al., 2011) as well as a pronounced substrate inhibition. With respect to the toxicity of bile salts as growth substrates, low *K*_m_ and high turnover of the substrate are obvious advantages for a fast alleviation of the toxic properties by transformation of the bile salts. The necessity for a fast detoxification mechanism in *Sphingobium* sp. strain Chol11 became obvious by exposition to high concentrations of cholate such as 10 mM leading to massive reduction of CFUs. For a fast response, 5β-Δ^4^-KSTD1 should be continuously present in *Sphingobium* sp. strain Chol11 cells. The fact that glucose-grown cells of *Sphingobium* sp. strain Chol11 were readily induced for transforming cholate into HOCDA (**11**) indicated that there is a basal expression of the respective genes for 3α-HSD, 5β-Δ^4^-KSTD, and 7α-hydroxysteroid dehydratase involved in this presumptive detoxification.

The phenotype of the deletion mutant showed that 5β-Δ^4^-KSTD1 was not essential for growth and cholate degradation and that isoenzymes must exist. However, especially the altered accumulation of 3-ketocholate (**5** in Figure 2) and Δ^4^-3-ketocholate (**6**) in mutant cultures indicated a prominent *in-vivo* role for 5β-Δ^4^-KSTD1. Additionally, it also showed that the isoenzymes need to be upregulated, which is further supported by the strongly delayed transformation of cholate into HOCDA (**11**) in the presence of the translation-blocking chloramphenicol. The minor effect of the deletion on growth with cholate together with the stronger effects on intermediate concentration indicate, that the first steps of degradation including generation of the Δ^4^-double bond do not determine velocity of bile-salt degradation. Thus, and corresponding with the kinetic parameters of 5β-Δ^4^-KSTD1, the first steps should proceed very fast, whereas following steps such as side-chain degradation could rate-determining. Kinetics of cholate degradation with the prominent transient accumulation of Δ^4^-3-ketocholate (**6**) and HOCDA (**11**) indicate that their further metabolization is a bottleneck in this catabolic pathway.

In contrast to high turnover and substrate specificity of 5β-Δ^4^-KSTD1, the advantage of substrate inhibition for growth with toxic bile salts is less obvious. While substrate inhibition is apparently a very widespread phenomenon, its physiological functions are mainly unknown but appear to be very diverse (Reed et al., 2010). These functions also comprise the homoeostasis of metabolic pathways. Activities of Hsh2 and CoA-ligase SclA, the enzymes following 5β-Δ^4^-KSTD1 in bile-salt degradation, are much lower compared to the very high activity of 5β-Δ^4^-KSTD1 (Yücel et al., 2016, 2018b). Thus, 5β-Δ^4^-KSTD1 product Δ^4^-3-ketocholate would accumulate in high amounts if 5β-Δ^4^-KSTD1 unrestrictedly functioned at highest activity. An overflow with Δ^4^-3-ketocholate could create a depletion of the pools of electron acceptors and CoA. Lacking substrate inhibition, 5β-Δ^4^-KSTD1 could lead to a redox imbalance by directly or indirectly reducing large amounts of cytochromes, NAD(P) or ubiquinone whenever high concentrations of bile salts resulting in high concentrations of 3-keto bile salts are present in the cell. Depletion of free CoA has been hypothesized to be the reason for toxicity of cholesterol in *Mycobacterium tuberculosis* mutants lacking genes involved in degradation of HIPs (**10** in Figure 2; Crowe et al., 2017). Presumably, HIP-CoA compounds accumulate in these strains binding large parts of the CoA-pool. In *Sphingobium* sp. strain Chol11, substrate inhibition of 5β-Δ^4^-KSTD1 would directly reduce CoA-requirement for Δ^4^-3-ketocholate and subsequent intermediate HOCDA, which are substrates for steroid-CoA ligase Scl1 (Yücel et al., 2018b). Based on these considerations, substrate inhibition by 3-ketocholate (**5** in Figure 2) can be interpreted as a circuit breaker of bile-salt metabolism.

Curbing or even shutting down bile-salt catabolism by substrate inhibition would create high levels of intracellular bile-salts and would also shut down growth. In agreement with that, *Sphingobium* sp. strain Chol11 showed delayed growth with 3 mM cholate and did not grow at cholate concentrations above 5 mM. Of course, this shutdown could also at least partly be due to toxic effects exerted by cholate. Therefore, the cells must be able to quickly reduce bile-salt levels by pumping them out of the cell. The existence of respective efflux pumps is strongly supported by the transient extracellular accumulation of 3-ketocholate, Δ^4^-3-ketocholate, and HOCDA in the culture supernatant. To date, no specific efflux pumps in bile-salt degrading bacteria have been identified. However, as the transient accumulation of intermediates of bile-salt degradation is a common phenomenon in the respective bacteria it appears justified to postulate such efflux pumps to be very important for enabling growth with these toxic substrates (Swain et al., 2012; Holert et al., 2014). In addition, further-downstream metabolites of cholate degradation might also have toxic effects on the cells, and substrate inhibition would therefore prevent the potential accumulation of these metabolites. Toxic effects are known for androsta-1,4-diene-3,17-diones (ADDs, such as 12β-DHADD, **8** in Figure 2; Perez et al., 2003; Philipp et al., 2006) and have also been postulated for other bacterial bile-salt degradation intermediates (Swain et al., 2012).

5β-Δ^4^-KSTD1 showed a relaxed substrate specificity regarding side chain and hydroxylation pattern for 5β-steroids including derivatives of all human bile salts. In contrast, it showed no activity with 5α-steroids having a flat conformation of the steroid skeleton. Although only two 5α-steroids could be tested due to their limited commercial availability, it was obvious that the specificity of 5β-Δ^4^-KSTD1 was dictated by the bent conformation of the steroid skeleton rather than the presence or absence of a carboxylic side chain. This conformation specificity is reflected by the low similarity to 5α-Δ^4^-KSTDs indicating a different phylogenetic origin.

5β-Δ^4^-KSTD1 as well as 5β-Δ^4^-KSTDs BaiCD and BaiH from *C. scindens* share their large size and co-factor composition and belong to the so-called thermophilic-like subgroup of Old Yellow Enzymes (Peters et al., 2019) as supported by phylogenetic analyses. For the 5β-Δ^4^-KSTD BaiCD, it was long speculated (Ridlon et al., 2006; Kang et al., 2008) and recently shown (Funabashi et al., 2020) that the preferred substrate is a CoA-bound 3-keto-bile salt. For 5β-Δ^4^-KSTD1 from *Sphingobium* sp. strain Chol11, it is unlikely that 3-ketocholyl-CoA (CoA-ester of **5** in Figure 2) is a substrate because the CoA-ligase adding CoA to C_5_ side chain of bile salts, SclA, strongly prefers Δ^4^-3-ketocholate (**6**) and HOCDA (**11**) over 3-ketocholate (Yücel et al., 2018b), indicating A-ring oxidation to occurs first. As the dehydrogenation of 3-ketocholate was possible with the artificial electron acceptors K_3_Fe(CN)_6_, PMS and DCPIP, quinones are likely to be natural electron acceptors of 5β-Δ^4^-KSTD1. This conclusion is in agreement with earlier enzymatic studies with cell extracts of *Sphingobium* sp. strain Chol11 (Yücel et al., 2016) and of *P. stutzeri* Chol1 (Birkenmaier et al., 2007) in which likewise no oxidation of 3-ketocholate was observed with NAD^+^ but rather the requirement of K_3_Fe(CN)_6_ or PMS was reported. In contrast, BaiCD from *C. scindens* uses NAD^+^ as electron acceptor. It has also been shown to catalyze the reverse reaction using NADH as electron donor (Kang et al., 2008; Funabashi et al., 2020). The same has been reported for BaiH, which has 3-ketoursodeoxycholate-oxidizing activity with NAD^+^ as co-electron acceptor, but also catalyzes the NADH-dependent reduction of a Δ^6^-double bond in intermediates such as HOCDA (**11**; Franklund et al., 1993; Kang et al., 2008). Generally, several Old Yellow Enzymes have been shown to reduce steroids (Williams and Bruce, 2002; Toogood et al., 2010). However, a reduction of Δ^4^-3-ketocholate by 5β-Δ^4^-KSTD1 with NAD(P)H could not be measured.

Based on the sequence, homologs of 5β-Δ^4^-KSTD could be found in known bile-salt degrading bacteria. However, the additional two homologs in *Sphingobium* sp. strain Chol11 did not exhibit any 5β-Δ^4^-KSTD activity toward any tested bile salt and could, therefore, not explain the phenotype of deletion mutant *Sphingobium* sp. strain Chol11 Δ*5β-Δ^4^-kstd1*. A possible explanation could be that these enzymes process CoA-esters of 3-ketobile salts which could not be provided in these assays. However, the aforementioned substrate specificity of the CoA-ligase contradicts this theory. Similarly, the only 5β-Δ^4^-KSTD1 homolog C211_11427 of *P. stutzeri* Chol1 did not show any activity toward any 3-keto-bile salt. It is unlikely that these genes could not functionally be expressed in *E. coli*, because several *Sphingobium* sp. strain Chol11 genes, including very similar *5β-Δ^4^-kstd1* itself, have been successfully expressed in this host (Yücel et al., 2016, 2018b). Additionally, a lacking phenotype of the deletion mutant of aforementioned candidate gene *c211_11427* from *P. stutzeri* Chol1 supported that this enzyme was not responsible for the dehydrogenation of 3-ketocholate in this bacterium. These results indicate that yet unknown alternate enzymes catalyzing this reaction must exist in some bile-salt degrading bacteria that differ significantly in their sequence and probably belong to a different protein family.

Of all tested homologs, only CasH from a steroid degradation cluster in *R. jostii* RHA1 was confirmed to be a 5β-Δ^4^-KSTD. CasH is encoded in a cluster of cholate degradation genes, in proximity to many *cas* genes coding for steroid carboxylic C_5_ side chain degradation enzymes (Mohn et al., 2012). As homologs of CasH and ACY32066.1 from *C. testosteroni* cannot be found in all steroid-degrading bacteria, it was hypothesized, that these are not associated with steroid degradation (Bergstrand et al., 2016). However, 5β-Δ^4^-KSTD1 homologs are only needed for degradation of a subset of steroids. Nevertheless, these include the major intestinal cholesterol transformation product coprostanol as well as many steroidal toxins.

The high efficiency and versatility of 5β-Δ^4^-KSTD1 as one factor for the ability of *Sphingobium* sp. strain Chol11 to transform a large number of bile salts as well as multiplicity of the respective degradation enzymes suggest that *Sphingobium* sp. strain Chol11 appears to be well-adapted to bile-salt containing environments. Additionally, bile-salt degradation is also observed with other type strains from this family (unpublished results). As Sphingomonads are not known as typical members of the microbiome of vertebrates the origin and the conservation of these properties is not obvious but certainly draws attention to the ecology and evolutionary history of this group of the α-proteobacteria.

## Data Availability Statement

The datasets presented in this study can be found in theonline repository PRIDE at: https://www.ebi.ac.uk/pride/archive/ with the accession number PXD022404.

## Author Contributions

FF conceptualized the study together with BP, planned and performed most of the experiments, wrote the first draft of the manuscript, and also supervised GM. GM constructed the deletion mutant and performed the corresponding experiments. LW performed proteome studies. SD performed protein purification, gel filtration, and helped with evaluations of enzyme assays. BP was involved in conceptualization of the study, planning of the experiments, and writing of the manuscript. All authors contributed to the manuscript revision and approved its submission.

### Conflict of Interest

The authors declare that the research was conducted in the absence of any commercial or financial relationships that could be construed as a potential conflict of interest.

## Abbreviations

ADD: Androsta-1,4-diene-3,17-dione
CFU: Colony forming unit
DCPIP: 2,6-Dichlorophenolindophenol
ESI: Electro-spray ion source
2D DIGE: Two-dimensional difference gel electrophoresis
HSD: Hydroxysteroid dehydrogenase
IPTG: Isopropyl-β-D-thiogalactopyranosid
KSTD: Ketosteroid dehydrogenase
LB: Lysogeny broth medium
OD_600_: Optical density at 600 nm
PMS: Phenazine methosulfate
SDS: Sodium dodecyl sulfate
SOE: Splicing by overlapping extension
12β-DHADD: 7,12β-Dihydroxy-androsta-1,4-diene-3,17-dione (**8** in Figure 2)
THSATD: 3,7,12-Trihydroxy-9,10-*seco*-androsta-1,3,5-triene-9,17-dione (**9**)
3αOH-HIP: 3aα-OH-4a(30-Propanoate)-7aβ-methylhexahydro-1-indanone (**10**)
HOCDA: 12-Hydroxy-3-oxo-chola-4,6-dienoate (**11**)
HATD: 12-Hydroxy-androsta-1,4,6-triene-3,12-dione (**12**)
DHSATD: 3,12-Dihydroxy-9,10-*seco*-androsta-1,3,5,6-tetraene-9,17-dione (**13**)
