# Supplementary material for: Substrate Inhibition of 5β-Δ4-3-Ketosteroid Dehydrogenase in Sphingobium sp. Strain Chol11 Acts as Circuit Breaker During Growth With Toxic Bile Salts
Source: Front Microbiol. 2021 Mar 23;12:655312. doi: 10.3389/fmicb.2021.655312 (PMC8044976; doi:10.3389/fmicb.2021.655312)
Supplement: Supplementary file 1 [file Data_Sheet_1.pdf]

Table of contents

Figure S1. SDS-PAGE of purified 5β-Δ^4-KSTD1 and aliquots from several steps of the purification process.

Figure S2. Gel filtration chromatography for determining size of 5β-Δ^4-KSTD1 using a marker with standards as indicated.

Figure S3. Enzyme assays with 3-ketocholate as substrate and $K_3Fe(CN)_6$ as electron acceptor. (**A**) MS-chromatogram in negative mode of an enzyme assay with purified 5β-Δ^4-KSTD1 and control without enzyme (**B**) MS-chromatogram in negative mode of an enzyme assay with only purified 5β-Δ^4-KSTD1 and with additional cell extract of *E. coli* MG1655 pBBR1MCS-5::*hsh2*.

Figure S4. Enzyme assays with 3-keto-bile salts as substrate, $K_3Fe(CN)_6$ as electron acceptor and cell extracts of *E. coli* MG1655 pBBR1MCS5 carrying genes for homologs of 5β-Δ^4-KSTD1. UV-chromatograms at 245 nm are displayed. Substrates are (**A**) 3-ketochenodeoxycholate, (**B**) 3-ketodeoxycholate, (**C**) 3-ketoursodeoxycholate and (**D**) 3-ketolithocholate.

Figure S5. Catalytic properties of 5β-Δ^4-KSTD1. (**A**) Activity of 5β-Δ^4-KSTD1 with varying concentrations of 5β-androstane-3,17-dione and 1 mM $K_3Fe(CN)_6$ at pH 7.8. (**B**) Influence of pH on enzyme activity of 5β-Δ^4-KSTD1 with 0.5 mM 3-ketocholate as substrate in Mc-Ilvaine buffer.

Figure S6. Sensitivity of *Sphingobium* sp. strain Chol11 wt and Δ*5β-Δ⁴-kstd1* towards cholate. (**A**) Growth of *Sphingobium* sp. strain Chol11 wt with different concentrations of cholate. (**B**) Growth of *Sphingobium* sp. strain Chol11 wt and Δ*5β-Δ^4-kstd1* with different concentrations of cholate. (**C+D**) Toxicity of cholate towards *Sphingobium* sp. strain Chol11 wt and Δ*5β-Δ^4-kstd1*. Remaining CFU in cell suspensions after incubation for 15 min (**C**) and 90 min (**D**) with given cholate concentrations.

Figure S7. Sequence of 5β-Δ^4-KSTD1 homolog from *Dietzia* sp. strain Chol2.

Figure S8. Alignment of genomic DNA of *Sphingobium* sp. strain Chol11 wt containing *5β-Δ^4-kstd1* and the sequenced part of genomic DNA of *Sphingobium* sp. strain Chol11 Δ*5β-Δ^4-kstd1*.

Table S1. Protein quantification data for 5β-Δ^4-KSTD1 during profiling of soluble proteins by 2D DIGE from cholate-grown cells when compared to glucose-grown cells as reference state.

Table S2. Protein identification data for 5β-Δ^4-KSTD1 during profiling of soluble proteins by 2D DIGE from cholate-grown cells when compared to glucose-grown cells as reference state.

Text S1. Profiling of soluble proteins by 2D DIGE and protein identification by MALDI-TOF-MS/MS.

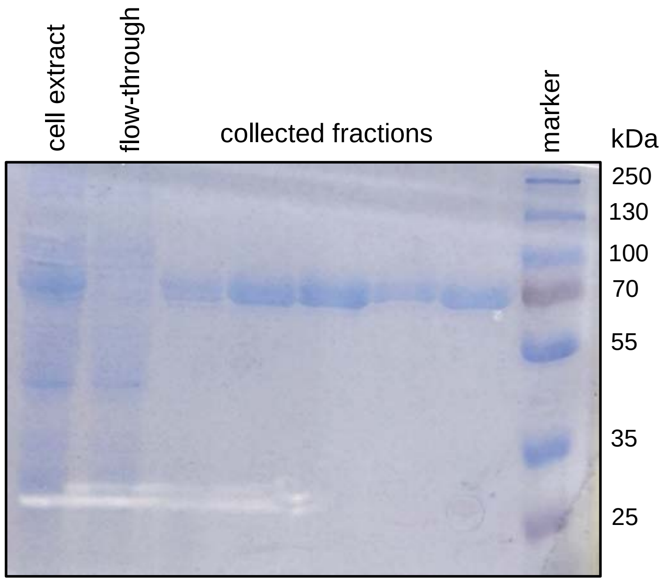

Figure S1. SDS-PAGE of purified 5β-Δ^4-KSTD1 and aliquots from several steps of the purification process. 5β-Δ^4-KSTD1 was his-tagged at both N- and C-termini. Flow-trough: discharge of FPLC, collected fractions: protein containing fractions eluted with increasing concentration of imidazole from left to right, shown fractions were pooled.

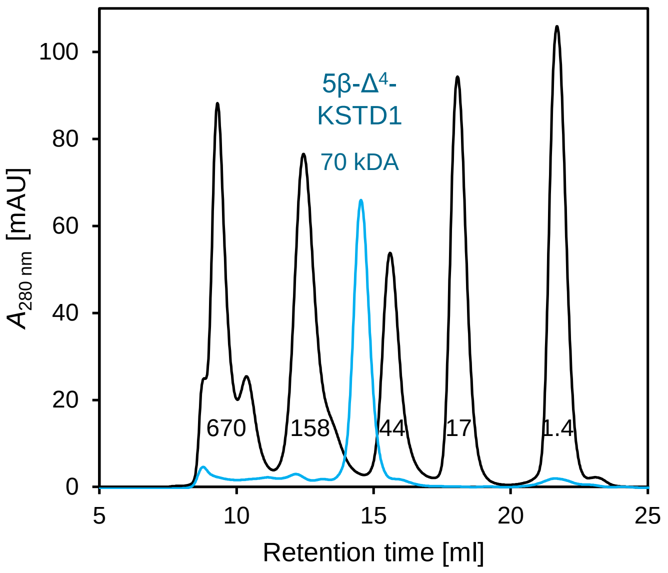

Figure S2. Gel filtration chromatography for determining size of 5β-Δ[4]-KSTD1 (blue) using a marker (black) with standards as indicated.

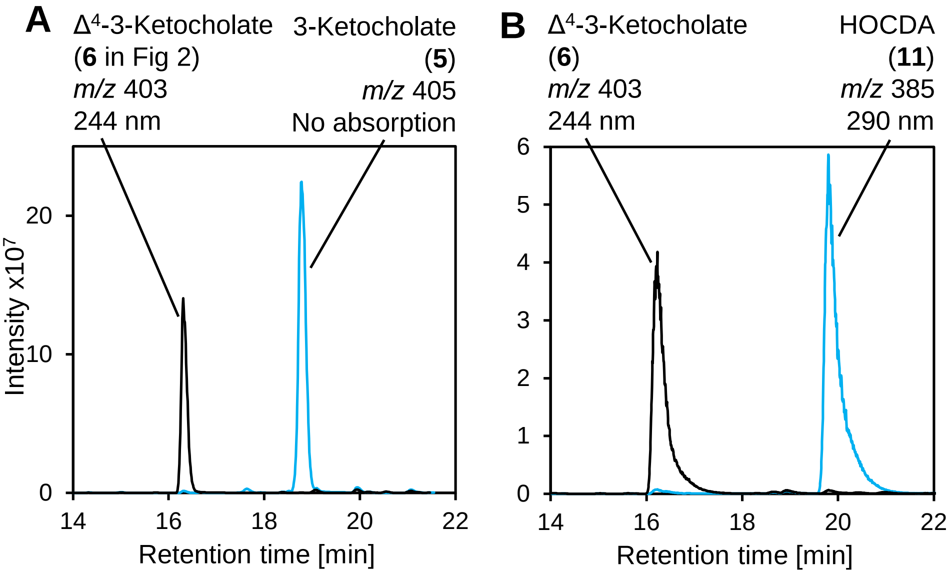

Figure S3. Enzyme assays with 3-ketocholate (**5** in Fig 2) as substrate and $K_3Fe(CN)_6$ as electron acceptor. (**A**) MS-chromatogram in negative mode of an enzyme assay with purified 5β-Δ^4-KSTD1 (black) and control without enzyme (blue) (**B**) MS-chromatogram in negative mode of an enzyme assay with only purified 5β-Δ^4-KSTD1 (black) and with additional cell extract of *E. coli* MG1655 pBBR1MCS-5::*hsh2* (black). Steroid compounds were identified due to retention time, absorbance spectrum and mass. Masses are indicated for the respective deprotonated acids.

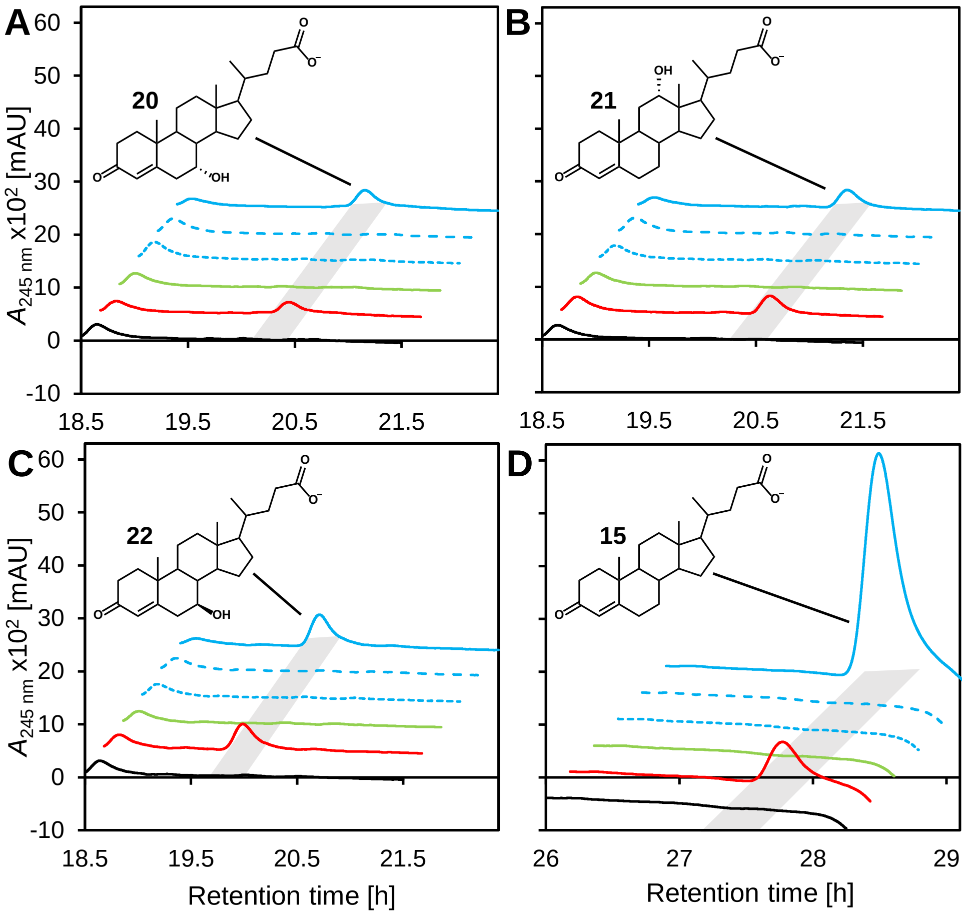

Figure S4. Enzyme assays with 3-keto-bile salts as substrate, $K_3Fe(CN)_6$ as electron acceptor and cell extracts of *E. coli* MG1655 pBBR1MCS5 carrying genes for homologs of 5β-Δ⁴-KSTD1. UV-chromatograms at 245 nm are displayed. Substrates are (**A**) 3-ketochenodeoxycholate, (**B**) 3-ketodeoxycholate, (**C**) 3-ketoursodeoxycholate and (**D**) 3-ketolithocholate. Continuous blue *5β-Δ⁴-kstd1*, dashed blue *nov2c085*, dotted blue *nov2c314*, all from *Sphingobium* sp. strain Chol11, green *c211_11427* from *P. stutzeri* Chol1, red *casH* from *R. jostii* RHA1, black empty vector control. Chromatograms are depicted with an offset in both retention time and intensity for easier distinction. Grey indicates retention time of the respective Δ⁴-3-keto-bile salt. Steroid compounds were identified due to retention time, absorbance spectrum and mass. **20**: Δ⁴-3-ketochenodeoxycholate, **21**: Δ⁴-3-ketodeoxycholate, **22**: Δ⁴-3-ketoursodeoxycholate.

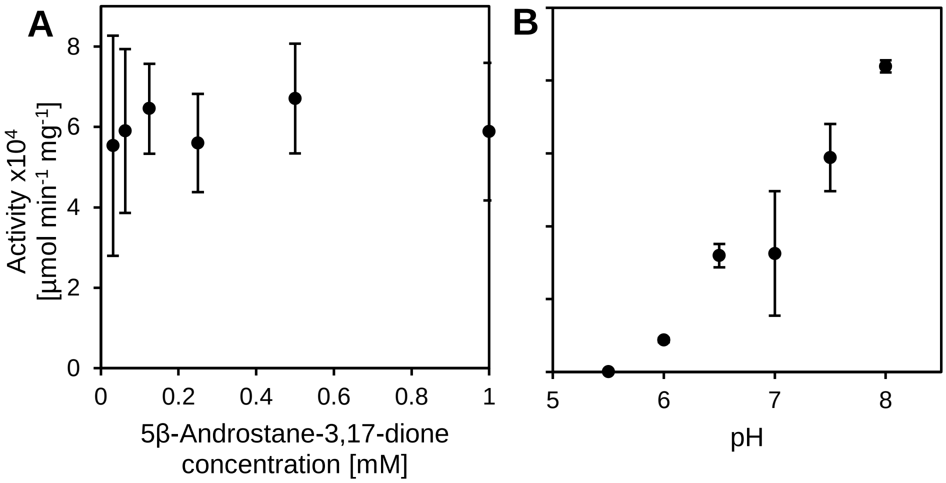

Figure S5. Catalytic properties of 5β-Δ^{4}-KSTD1. (**A**) Activity of 5β-Δ^{4}-KSTD1 with varying concentrations of 5β-androstane-3,17-dione (**16** in Fig 6) and 1 mM $K_3Fe(CN)_6$ at pH 7.8. (**B**) Influence of pH on enzyme activity of 5β-Δ^{4}-KSTD1 with 0.5 mM 3-ketocholate as substrate (**5** in Fig S1) in Mc-Ilvaine buffer. Error bars indicate standard deviation, which may not be visible if too small (n=3).

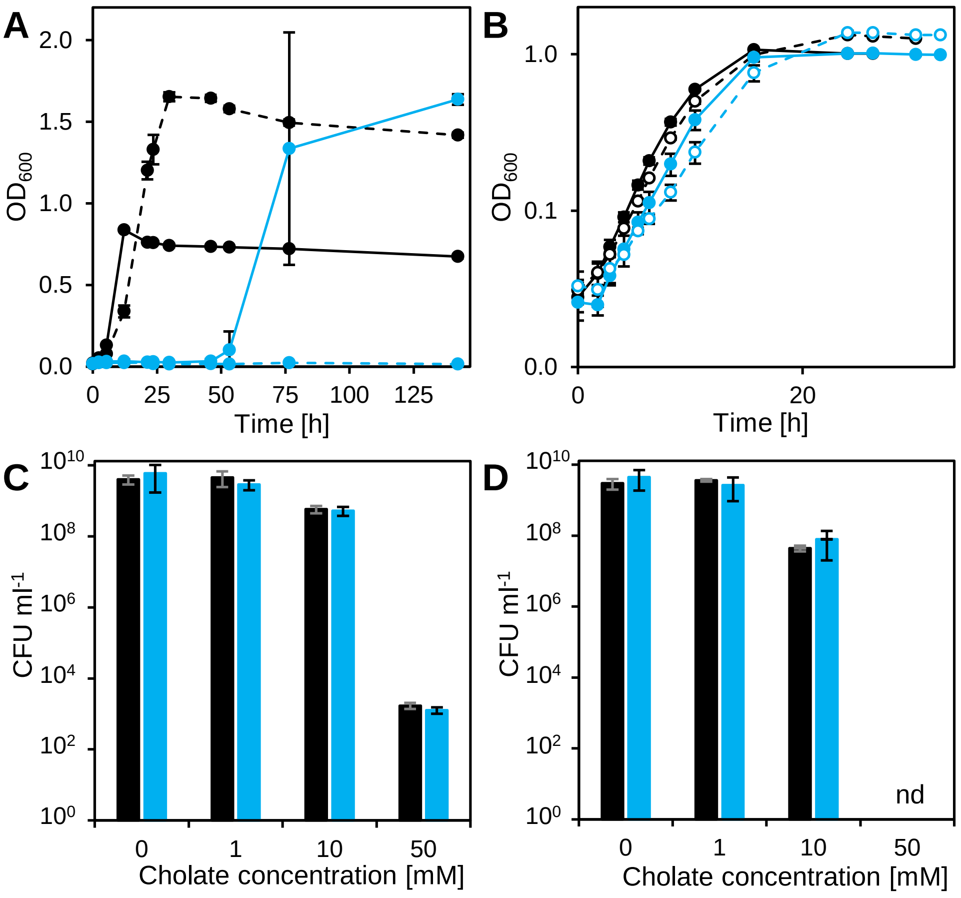

Figure S6. Sensitivity of *Sphingobium* sp. strain Chol11 wt and Δ*5β-Δ*^{4}*-kstd1* towards cholate. (**A**) Growth of *Sphingobium* sp. strain Chol11 wt with different concentrations of cholate. Black solid line 1 mM cholate, black dashed line 3 mM cholate, blue solid line 4 mM cholate, blue dashed line 5 mM cholate. (**B**) Growth of *Sphingobium* sp. strain Chol11 wt (black) and Δ*5β-Δ*^{4}*-kstd1* (blue) with 2 mM cholate (solid lines) and 3 mM cholate (dashed lines). (**C+D**) Toxicity of cholate towards *Sphingobium* sp. strain Chol11 wt (black) and Δ*5β-Δ*^{4}*-kstd1* (blue). Remaining CFU in cell suspensions after incubation for 15 min (**C**) and 90 min (**D**) with given cholate concentrations. nd: not detectable, error bars indicate standard deviation and may not be visible if to small (n=3).

> *Dietzia* sp. strain Chol2, protein 2765
MTTLTSFPRLLSPGRIGPMSTANRIVLPAMDMNVSEDGEIEQREIDHYVARAAGGAGLIITGACAVAFPHGAASMKEPGLSDD
RFLPGLTALADAVHAAGSKLCVQSTHHGKVARVDIANDRPVIAPSAPDYDYDYSALADSTPDELGRMGAATGGKKTVYREMTG
EDIAWLVSTWADAADRIARSGADAIEIHVAHGYILGVFLNRRDNQRTDAYGGSLTNRARLACEVIAAVKERVGDTLAVLVRVS
GEEYGQDGGLTLEEAIEASRMFERAGADAIHVTGWGRNPFDNFTDGPLPDTVGAYLDNAAAIKQAVSVPVIAVGRMLPEVAEK
AIDDDKVDFAAMGRQLLADPELPNKLAAGTPELVRPCINCYLCVAENFFDDTPFCAVNPALGNETLLPLTPAPARTHVVVVGA
GPAGLEAARVLAERGHRVTVVDKSDRLGGTMWFSSLTTPDNEPLIRWFAAEIDRLGIEVRLNTEATPESLRALQPDWVVVATG
AVRPAPQIPGGDLPHVHTGDSLRATMLGTATADEAGLALRVIGRLGRLSGITKSPSLIRRLTRFALPMGKNVVVIGGSLVGLE
LAEFLAERGRRVTLLHDGQQLGLPLAMPRRWTAVRRAREHDVAIHRRVTVTRITESDVQWTDRKGAAASAPADMVIYADGTTA
SAPLADQLREAGVEVEVIGDAGEVGYIHGAIHSAWGVATVG

Figure S7. Sequence of 5β-Δ^4-KSTD1 homolog from *Dietzia* sp. strain Chol2.

```
gDNA_wt     1     CTATTATGACGCCGTGCAATCGCCGAACGTAGAGGTGGAGCGGAATCGCATCGCCCGCGT  60

gDNA_wt     61    CGAGGCCAAGGGCGTGCGGATGGAAGACGGGCGGCTGCACGAACTTGATACGCTGGTGCT  120
                           |||||||||||||||||||||||||||||||||||||||||||||||||||
seq_dkstd1  1              GGGCGTGCGGATGGAAGACGGGCGGCTGCACGAACTTGATACGCTGGTGCT  51

gDNA_wt     121   GGCGACCGGCTTCCATGCGGACCGCTTCATCCGCCCGACAACGGTTCTGGGCCGGGGTGG  180
                  ||||||||||||||||||||||||||||||||||||||||||||||||||||||||||||
seq_dkstd1  52    GGCGACCGGCTTCCATGCGGACCGCTTCATCCGCCCGACAACGGTTCTGGGCCGGGGTGG  111

gDNA_wt     181   CGTTTCGCTCGATGATATATGGTCTGTTCGTCCGACGGCCTATTATGCCGTGACCATCCC  240
                  ||||||||||||||||||||||||||||||||||||||||||||||||||||||||||||
seq_dkstd1  112   CGTTTCGCTCGATGATATATGGTCTGTTCGTCCGACGGCCTATTATGCCGTGACCATCCC  171

gDNA_wt     241   GGGCTTTCCGAACTTCTTCATGTTGAATGGTCCGACCGGTCCTGTCGGCAACTTCTCCCT  300
                  ||||||||||||||||||||||||||||||||||||||||||||||||||||||||||||
seq_dkstd1  172   GGGCTTTCCGAACTTCTTCATGTTGAATGGTCCGACCGGTCCTGTCGGCAACTTCTCCCT  231

gDNA_wt     301   GATCGACATTGCGGAGAAGCAATGGGCATATGTGGATCAGCTGATCGATCTGCTGCGGTC  360
                  ||||||||||||||||||||||||||||||||||||||||||||||||||||||||||||
seq_dkstd1  232   GATCGACATTGCGGAGAAGCAATGGGCATATGTGGATCAGCTGATCGATCTGCTGCGGTC  291

gDNA_wt     361   GGGTGAGGCCCGGACGATCGAACCGAGCGCTGACGCTCACGCCGATTATGAGGAGCGGCG  420
                  ||||||||||||||||||||||||||||||||||||||||||||||||||||||||||||
seq_dkstd1  292   GGGTGAGGCCCGGACGATCGAACCGAGCGCTGACGCTCACGCCGATTATGAGGAGCGGCG  351

gDNA_wt     421   GATTGCGGCGGCGCGTACGACGATCTTCGGATCGGGTTGCAACAGCTGGTATCTGGATGC  480
                  ||||||||||||||||||||||||||||||||||||||||||||||||||||||||||||
seq_dkstd1  352   GATTGCGGCGGCGCGTACGACGATCTTCGGATCGGGTTGCAACAGCTGGTATCTGGATGC  411

gDNA_wt     481   CACCGGCGTTCCGGCAAGCTGGCCCTGGAGCTATGAGGCATTTGCCGATGCCATGGCGGC  540
                  ||||||||||||||||||||||||||||||||||||||||||||||||||||||||||||
seq_dkstd1  412   CACCGGCGTTCCGGCAAGCTGGCCCTGGAGCTATGAGGCATTTGCCGATGCCATGGCGGC  471

gDNA_wt     541   TCCGGTGCTGACGGATTATCGAGTTGAGGCTGCGGCTGAACTGGTTGAGCGGTGAGGGGT  600
                  ||||||||||||||||||||||||||||||||||||||||||||||||||||||||||||
seq_dkstd1  472   TCCGGTGCTGACGGATTATCGAGTTGAGGCTGCGGCTGAACTGGTTGAGCGGTGAGGGGT  531

gDNA_wt     601   TTTTAATCCCTCAAATCCGTTCGGACTGAGTTTGTCGAAGCCCCTCACTGCATAAAGAAG  660
                  ||||||||||||||||||||||||||||||||||||||||||||||||||||||||||||
seq_dkstd1  532   TTTTAATCCCTCAAATCCGTTCGGACTGAGTTTGTCGAAGCCCCTCACTGCATAAAGAAG  591

gDNA_wt     661   TAAGGCCCTTCGACAGGCTCAGGGCGAACGGTTGTTGGGTGGTCTCGATAAGGGGCAGCT  720
                  ||||||||||||||||||||||||||||||||||||||||||||||||||||||||||||
seq_dkstd1  592   TAAGGCCCTTCGACAGGCTCAGGGCGAACGGTTGTTGGGTGGTCTCGATAAGGGGCAGCT  651

gDNA_wt     721   CAGATACAAAAAGGGGAGCGTTGCGGCGCTCCCCTTTTCTTTGGCCTTGAAGATCAGATC  780
                  ||||||||||||||||||||||||||||||||||||||||||||||||||||||||||
seq_dkstd1  652   CAGATACAAAAAGGGGAGCGTTGCGGCGCTCCCCTTTTCTTTGGCCTTGAAGATCAGA--  709

gDNA_wt     781   AGCGGCCCATGGCGGCCACGGCTTCGGCTGCACCACGGATCGCGCCTTCGATATAGGTGA  840

seq_dkstd1        ------------------------------------------------------------

gDNA_wt     841   CGCCGGTGCCGTCGCCGACCTCCACCACATCATGGCCCTGTGCGCGCAGGGCATTGGCAA  900

seq_dkstd1        ------------------------------------------------------------

gDNA_wt     901   GGGTGGAGTCGCCATGCGCGCCCATGGCAACCACCACATGATCGGCAGCAACGCTCTTCT  960

seq_dkstd1        ------------------------------------------------------------

gDNA_wt     961   GCTCACCGTCTGCACTGGTGAAGGTGACGCTGTTCTTGCCGATCGCGATGCCGCTCGCGC  1020

seq_dkstd1        ------------------------------------------------------------

gDNA_wt     1021  CGGGGAACATGGCCACGCCATGTTCGCGCAGTTCCGCCAGCAGGCGCATGCGGCGCACCA  1080

seq_dkstd1        ------------------------------------------------------------

gDNA_wt     1081  GCAGCAGGCCGCCGCCGAACCTGGGCGCTTCGCCGACAACCGTAACTTCACGGCCGCGTT  1140

seq_dkstd1        ------------------------------------------------------------

gDNA_wt     1141  CCATCAGGAATTCGGCCAGCTCAACGCCGACCAACTCGCCGCCGATGATCACGACGCGTT  1200

seq_dkstd1        ------------------------------------------------------------

gDNA_wt     1201  TACCGAGCGGCATCCAGGCATGGGTCGCCTTGCGCACCAGGTCGAGATTGGCGGTTGCGC  1260

seq_dkstd1        ------------------------------------------------------------

gDNA_wt     1261  CGGTCGCTGCGCCGAGCTTCGTCGCGACGCGGGTGGTCCAGCTGGTCTTGCGACGGATTT  1320

seq_dkstd1        ------------------------------------------------------------

gDNA_wt     1321  CAGCCGAATCCTCGCCCAGCATCATGCCGCGCAGATCGTCGCCCGACAGCACGAAGTCCT  1380

seq_dkstd1        ------------------------------------------------------------

gDNA_wt     1381  GATCATTGCCGGGGATCGGCGGCATGTCGCGCACGGCGCCGGTCGCGACGATGACCTTGT  1440

seq_dkstd1        ------------------------------------------------------------

gDNA_wt     1441  CGGGCTTGAGGCTGGCGATGAGGTCCGGGGTCGCGACAGTCTTCAGGCGCACATCGACCT  1500

seq_dkstd1        ------------------------------------------------------------

gDNA_wt     1501  TCGACTGGCCGATCTGGTCACGCAGCCAGTTGAGCAGCCGCTCATTGGGCTCATAAGCGA  1560

seq_dkstd1        ------------------------------------------------------------

gDNA_wt     1561  GGCCGGCGAAGCGCAGCGTGCCGCCCAGGCGATCGGATTTTTCGAGAAGCGTGACCTTCG  1620

seq_dkstd1        ------------------------------------------------------------

gDNA_wt     1621  CGCCCGCAAGGTCCAGCCTGCGCGCGGCTTCCATGCCGCCGGGGCCGCCGCCGATAACCA  1680

seq_dkstd1        ------------------------------------------------------------

gDNA_wt     1681  CGACATGGCCGGGAACCTTGGCCGGGGACCAGTCGAGATCCTCATAACCGGTTTCCGACC  1740

seq_dkstd1        ------------------------------------------------------------

gDNA_wt     1741  GCACGGCGCAGCGCACCTGTTCGCGAAGATAGGCGGTGCTGACGCAGGTGTAGCAATAGA  1800

seq_dkstd1        ------------------------------------------------------------

gDNA_wt     1801  TGCACGGGCGGATATCGGCCACCCGGCCTTCGGTCAGCTTGCGGGGCAGATCAGGGTCGG  1860

seq_dkstd1        ------------------------------------------------------------

gDNA_wt     1861  CGAGGAGCTTGCGACCCATGGCAAGGAAGTCGAACTCGCCCTGCGCCAACCCCTTGTCGC  1920

seq_dkstd1        ------------------------------------------------------------

gDNA_wt     1921  CGCGATCGAGCTCGACCCGACCCGACGCGATCACGGGGATCGAAACCACCTTCCGGATTG  1980

seq_dkstd1        ------------------------------------------------------------

gDNA_wt     1981  CGGCGGCGGCTTCCAGGTTGAAGGCGGGTTCATGCGGGATGTTGGAACCCGAATGGAGCT  2040

seq_dkstd1        ------------------------------------------------------------

gDNA_wt     2041  TGCCCTGGCCGACGTCATGATAGGCAGTGACGGTAAGCGCGTCGGCACCGGCCGCTTCGA  2100

seq_dkstd1        ------------------------------------------------------------

gDNA_wt     2101  TCATAGGCGCCAGTTCAAGCGCATGGTCGATGGTGATGCCGTTTTTGCCCAGCTCGCGTG  2160

seq_dkstd1        ------------------------------------------------------------

gDNA_wt     2161  AATCCATCTTCACCCAGACGGGGAAATCCGGGCCGACCGCGGCGCGCACCGCGCGTACCA  2220

seq_dkstd1        ------------------------------------------------------------

gDNA_wt     2221  CATCGAGGAGCAGGCGCGCGCGACCTGCCACGTCGCCGCCATATTCATCCTCGCGCGTGT  2280

seq_dkstd1        ------------------------------------------------------------

gDNA_wt     2281  TGGTGTAGGGCGAAACGAAAGAGGAGAGCAGATAGCCGTGGCCACCGTGGATCTCCAGGC  2340

seq_dkstd1        ------------------------------------------------------------

gDNA_wt     2341  CGTCCATTCCGGCGCGCTTGGCGCGTAGCGCGGCGTCGGCGAACTGGCGGATCGCATGAT  2400

seq_dkstd1        ------------------------------------------------------------

gDNA_wt     2401  CGATGTCGGCCTTCTCCAGCACCTTGATCTGCGGCACCACGCCTCCGGCGAACGCAGCCA  2460

seq_dkstd1        ------------------------------------------------------------

gDNA_wt     2461  TCTCTTCGGGCAGGAAAAAGTCTGGGAAGTCGCCCGCGAAGGGGGGCGGATATGCCGGAG  2520

seq_dkstd1        ------------------------------------------------------------

gDNA_wt     2521  CCCAGAGAGGATGCCCATCCTGCGCGCTGTGCCCGGCGACAAGGCCGCCATGGTGAAGCT  2580

seq_dkstd1        ------------------------------------------------------------

gDNA_wt     2581  GAGCAGCGATGCGCCCGCCTGCTGCATGGACGGCCTCTGTCAGCTTCGTCAGGCCGGGCA  2640

seq_dkstd1        ------------------------------------------------------------

gDNA_wt     2641  GGAAGCGATCGTCCGAGATGGCGGTCTGGTTTGGCTGGACGGCGCCCACAGGCCAGGCGA  2700

seq_dkstd1        ------------------------------------------------------------

gDNA_wt     2701  CGCCGGTCACGCCCATGATGATGAGGCCAGCGCCGCCCTTCGCCTGGGCGACATGATAGT  2760

seq_dkstd1        ------------------------------------------------------------

gDNA_wt     2761  CGATAAGGCGCTGGCCGACCGTACCGTCGGCTTCTGCCAGGCTGACGCCCATGGCGGTGA  2820

seq_dkstd1        ------------------------------------------------------------

gDNA_wt     2821  CGATGATGCGGTTGGGAAGGGTGAGCATACCGATCTGGCCGGGCGTGGTGAGAGATGCAG  2880

seq_dkstd1        ------------------------------------------------------------

gDNA_wt     2881  GCGCTGCGCCCGAACGGGATTGTGAAAGGGCAGGGGCAGCAGTCATGAGAGTCTCCTAAT  2940
                                                                ||||||||||||||
seq_dkstd1  710   ----------------------------------------------GAGAGTCTCCTAAT  723

gDNA_wt     2941  TCGCCACATGCGTATGGTCTGCTCGTCTTTTACCGCCTTGTTCACTGTTAGTCAGGCGAA  3000
                  ||||||||||||||||||||||||||||||||||||||||||||||||||||||||||||
seq_dkstd1  724   TCGCCACATGCGTATGGTCTGCTCGTCTTTTACCGCCTTGTTCACTGTTAGTCAGGCGAA  783

gDNA_wt     3001  AATAACGGCGATCGCGTAAGAATTTGTACGATTCTTTCCCGATTGATCGATTCGCAAAAG  3060
                  ||||||||||||||||||||||||||||||||||||||||||||||||||||||||||
seq_dkstd1  784   AATAACGGCGATCGCGTAAGAATTTGTACGATTCTTTCCCGATTGATCGATTCGCAAA    841
```

Figure S8. Alignment of genomic DNA of *Sphingobium* sp. strain Chol11 wt containing *5β-Δ^4-kstd1* and the sequenced part of genomic DNA of *Sphingobium* sp. strain Chol11 Δ*5β-Δ^4-kstd1*, red: *5β-Δ^4-kstd1*, green: adjacent genes

Table S1. Protein quantification data for 5β-Δ^4-KSTD1 during profiling of soluble proteins by 2D DIGE from cholate-grown cells when compared to glucose-grown cells as reference state.

Anova p-value	Fold-change
8.66^{-9}	5.5

Table S2. Protein identification data for 5β-Δ^4-KSTD1 during profiling of soluble proteins by 2D DIGE from cholate-grown cells when compared to glucose-grown cells as reference state. Pept number of detected peptides, SC (%) sequence coverage.

	**PMF (MS)**			**PFF (MS/MS)**		
**Replicate**	Score	Pept	SC (%)	Score	Pept	SC (%)
**1**	224.0	19	22.0	138.1	2	4.2
**2**	268.0	26	35.8	630.4	7	18.9
**3**	339.0	36	46.0	569.6	6	12.2

Text S1. Profiling of soluble proteins by 2D DIGE and protein identification by MALDI-TOF-MS/MS.

For profiling of soluble proteins by means of 2D DIGE, proteins extracts of 4 biological replicate cultures per growth condition (i.e. cholate- and glucose-adapted cells) were prepared and labelled using 200 picomoles of Lightning SciDye DIGE fluors (SERVA, Heidelberg, Germany) as described [1] with cholate as test and glucose as reference state. First dimension isoelectric focusing and second dimension SDS-PAGE were performed as reported [1] and resulting gels digitalized using a CCD camera system (Intas Advanced 2D Imager; Intas Science Imaging Instruments GmbH, Göttingen, Germany). Cropped gel images were analyzed with the SameSpots™ software (version 5.0.1.0; TotalLab, Newcastle upon Tyne, UK) as described by Schnaars et al [2]. Spots of interest were excised and subjected to MALDI-TOF MS(/MS) analysis as reported [3]. Obtained mass spectra were used for searching against the translated genome of *Sphingobium* sp. strain Chol11 using a Mascot-server (version 2.3; Matrix Science, London, UK) and a mass tolerance of 25 ppm and 100 ppm for MS and MS/MS searches, respectively. For both, MS and MS/MS searches, Mascot scores not meeting the 95% certainty criterion were not considered significant. A single missed cleavage was allowed (enzyme trypsin) and carbamidomethyl (C) and oxidation (M) were set as fixed and variable modifications, respectively. The mass spectrometry proteomics data have been deposited to the ProteomeXchange Consortium via the PRIDE [4] partner repository with the dataset identifier PXD022404.

[1] Gade, D., Thiermann, J., Markowsky, D., and Rabus, R. (2003) Evaluation of two-dimensional difference gel electrophoresis for protein profiling. *J. Mol. Microbiol. Biotechnol.* **5**, 240–251

[2] Schnaars, V., Dörries, M., Hutchins, M., Wöhlbrand, L., and Rabus, R. (2018) What's the difference? 2D DIGE image analysis by DeCyder™ versus SameSpots™. *J. Mol. Microbiol. Biotechnol.* **28**, 128–136

[3] Zech, H., Hensler, M., Koßmehl, S., Drüppel, K., Wöhlbrand, L., Trautwein, K., Hulsch, R., Maschmann, U., Colby, T., Schmidt, J., Reinhardt, R., Schmidt-Hohagen, K., Schomburg, D., and Rabus, R. (2013) Adaptation of *Phaeobacter inhibens* DSM 17395 to growth with complex nutrients. *Proteomics*. **13**, 2851–2868

[4] Perez-Riverol, Y., Csordas, A., Bai, J., Bernal-Llinares, M., Hewapathirana, S., Kundu, D. J., Inuganti, A., Griss, J., Mayer, G., Eisenacher, M., Pérez, E., Uszkoreit, J., Pfeuffer, J., Sachsenberg, T., Yilmaz, Ş., Tiwary, S., Cox, J., Audain, E., Walzer, M., Jarnuczak, A. F., Ternent, T., Brazma, A., and Vizcaíno, J. A. (2019) The PRIDE database and related tools and resources in 2019: improving support for quantification data. *Nucleic Acids Res.* **47**, D442–D450
